# Temperature-Dependent Influence of FliA Overexpression on PHL628 *E. coli* Biofilm Growth and Composition

**DOI:** 10.3389/fcimb.2021.775270

**Published:** 2021-12-17

**Authors:** Luke D. Buck, Maddison M. Paladino, Kyogo Nagashima, Emma R. Brezel, Joshua S. Holtzman, Sarel J. Urso, Lisa M. Ryno

**Affiliations:** Department of Chemistry and Biochemistry, Oberlin College, Oberlin, OH, United States

**Keywords:** biofilm, FliA, confocal microscopy, *E coli*, extracellular matrix

## Abstract

Biofilm growth and survival pose a problem in both medical and industrial fields. Bacteria in biofilms are more tolerant to antibiotic treatment due to the inability of antibiotics to permeate to the bottom layers of cells in a biofilm and the creation of altered microenvironments of bacteria deep within the biofilm. Despite the abundance of information we have about *E. coli *biofilm growth and maturation, we are still learning how manipulating different signaling pathways influences the formation and fitness of biofilm. Understanding the impact of signaling pathways on biofilm formation may narrow the search for novel small molecule inhibitors or activators that affect biofilm production and stability. Here, we study the influence of the minor sigma transcription factor FliA (RpoF, sigma-28), which controls late-stage flagellar assembly and chemotaxis, on biofilm production and composition at various temperatures in the *E. coli* strain PHL628, which abundantly produces the extracellular structural protein curli. We examined FliA’s influence on external cellular structures like curli and flagella and the biomolecular composition of the biofilm’s extracellular polymeric substance (EPS) using biochemical assays, immunoblotting, and confocal laser scanning microscopy (CLSM). At 37°C, FliA overexpression results in the dramatic growth of biofilm in polystyrene plates and more modest yet significant biofilm growth on silica slides. We observed no significant differences in curli concentration and carbohydrate concentration in the EPS with FliA overexpression. Still, we did see significant changes in the abundance of EPS protein using CLSM at higher growth temperatures. We also noticed increased flagellin concentration, a major structural protein in flagella, occurred with FliA overexpression, specifically in planktonic cultures. These experiments have aided in narrowing our focus to FliA’s role in changing the protein composition of the EPS, which we will examine in future endeavors.

## 1 Introduction

Bacterial biofilms, communities of bacteria layered in a self-produced matrix of extracellular polymeric substances, are a pervasive form of life; more than 99.9% of bacteria can form biofilm (Donlan and Costerton, 2002; Lewandowski and Beyenak, 2013). Bacteria enter the sessile state for a number of reasons, all of which fall under the broad category of increasing an organism’s overall chance of survival. Biofilm protects bacterial cells against different forms of environmental stress such as nutrient deprivation, pH changes, oxygen radicals, disinfectants, and antibiotics (Jefferson, 2004). The extracellular polymeric substance (EPS), a thick matrix surrounding the cells in the biofilm, and their multilayer nature are the primary mechanisms of protection for the individual bacterial cells within a biofilm. Although the EPS is made up of 97% water, it is also composed of exopolysaccharides, polymers, proteins, nucleic acids, lipids, absorbed nutrients, and metabolites (Beloin et al., 2008). The EPS can bind and trap antimicrobials before they reach the individual cells. For example, negatively charged extracellular polysaccharides inhibit the diffusion of positively charged aminoglycoside antibiotics. The presence of the EPS can provide a physical barrier that slows the diffusion of antimicrobials to cells within the biofilm, which allows enzymes time to degrade certain toxic molecules (Lewis, 2001). Additionally, the metabolic pathways of biofilm cells are altered to make them more resistant to starvation and small molecule insults (Costerton et al., 1999; Stewart and Costerton, 2001).

One of biofilm’s most adaptive features is its role in antibiotic resistance. Antibiotic resistance is a serious global health concern and its costly effects makes studying bacterial biofilm an important topic of research. Biofilm serves as a protective mechanism against antimicrobial and disinfectant agents as a means to improve the bacteria’s biological fitness. In comparison to a free-swimming planktonic cell, maximally resistant biofilm bacteria in their stationary phase are 15-times more resistant to antimicrobial agents than planktonic cells (Schwank et al., 1998; Patel, 2005). Although the exact mechanism of how biofilm survives treatment with antibiotics is not yet comprehensively understood, a combination of factors is hypothesized to contribute to this adaptive tolerance mechanism. One explanation is the failure of antimicrobial agents to penetrate the biofilm matrix. The complex, three-dimensional structure of the biofilm matrix limits the diffusion of antimicrobial agents from reaching the bacterial cells in the biofilm community. Nutrient limitation also increases antimicrobial resistance by slowing the growth of bacteria (Stewart, 2015). Another newly emerging possibility to explain biofilm antibiotic resistance lies in the biofilm’s membrane protein composition. Previous research has suggested that altering the membrane protein composition of biofilm affects the bacteria’s intrinsic antimicrobial resistance by decreasing permeability of the cell to antimicrobial agents (Singh et al., 2017). This ubiquitous ability of microbes to form biofilm and its multi-layered defense mechanism for drug resistance makes biofilm research a complex and attractive area of study.

Biofilms form on almost any abiotic surface, in addition to being able to form both on and inside organic matter. One of the most favorable places for biofilms to attach, whether on an abiotic or biotic surface, is proximal to the air-liquid interface (Karatan and Watnick, 2009). A possible explanation for biofilm formation being localized in this way is the increased availability of oxygen at air-liquid interfaces as compared to locations completely submerged in the liquid (Wijman et al., 2007). Some strains of *E. coli* form difficult-to-eradicate biofilms that are responsible for nosocomial infections that originate on catheters, prostheses like prosthetic heart valves, cardiac pacemakers, and replacement joints that result in morbidity and mortality (Lindsay and Holy, 2006; Reisner et al., 2014). Studying the mechanisms involved in bacterial biofilm formation is important in order to enhance the effects of currently existing antibiotics as well as to identify new drug targets that can disable biofilm formation.

As a scientific community, we have a detailed understanding of the physical nature of the growth, maturation and dispersal of biofilm for a number of different organisms, but we still are learning about how the differential expression of genes can influence biofilm growth and composition (Prigent-Combaret et al., 2000; Jackson et al., 2002; O’Toole et al., 2003; Ren et al., 2004; Guttenplan and Kearns, 2013; Mika and Hengge, 2013; Zhang et al., 2013; Liu et al., 2014; Sharma et al., 2016). Biofilm formation occurs in a stepwise fashion; steps include initial reversible attachment, irreversible attachment, maturation, and dispersion. In order for *Escherichia coli* cells to form a biofilm, they must first contact a surface. In gram-negative bacteria like *E. coli*, whip-like appendages called flagella give the bacterial cells the motility necessary to move towards a surface and overcome the repulsive electrostatic and hydrodynamic forces near that surface (Belas, 2014; Chaban et al., 2015; Friedlander et al., 2015). Once the *E. coli* cells have made initial contact with the surface, the bacteria use their flagella to distribute over the surface and form the initial biofilm layer. Although useful in the initial biofilm-forming steps, flagella are not a requirement for biofilm formation; nonmotile *E. coli* can still form biofilms if they overexpress extracellular adhesion factors like curli fimbriae and pili (Karatan and Watnick, 2009; Wolska et al., 2016).

The master regulator of the transcriptional network that leads to functional flagella and motility is the transcription factor dual regulator FlhDC. FlhD and FlhC are proteins that form a heterohexameric complex that activates the transcription of several genes important for early flagellar assembly, in addition to activating the transcription of the minor sigma factor FliA (RpoF, sigma28). FliA is a transcription factor that affects its own downstream regulon, which codes for genes important in late-stage flagellar assembly and chemotaxis (Mytelka and Chamberlin, 1996; Fitzgerald et al., 2014). Some flagellar genes are subject to dual regulation by FlhDC and FliA, however the absence of FliA does not have a dramatic effect on dual-regulated genes (Fitzgerald et al., 2014). This indicates that FlhDC is the primary regulator necessary for the transcription of the main flagellar components, while FliA almost exclusively regulates genes involved in later stages of flagellar assembly (Fitzgerald et al., 2014). FliA also activates the transcription of several genes not related to flagella, including *yhjH*, a phosphodiesterase that cleaves bis-(3’-5’)-cyclic-diguanosine monophosphate (c-di-GMP) and has been implicated in biofilm formation through development of type I pili (Claret et al., 2007; Pesavento et al., 2008).

Previously, the importance of FliA and its influence on biofilm formation has been demonstrated in K-12 strains of *E. coli* that contain the derepressed R1*drd*19 conjugative plasmid, a plasmid that can be transformed into K-12 *E. coli* to induce robust biofilm formation (Reisner et al., 2003; Barrios et al., 2006). In these cells, if the regulator proteins Hha and YbaJ are deleted, less biofilm is formed and the cells increase the expression of FlhD and FliA, leading to greater motility. Interestingly, deletion of *fliA* in K-12 strains of *E. coli* that lack the R1*drd*19 conjugative plasmid *increase* biofilm formation, demonstrating that under certain genetic and environmental conditions, the motility of bacteria is indeed important for biofilm formation and growth, but is subject to complex regulation.

Here, we explore the influence of FliA overexpression on the PHL628 strain of *E. coli*, which is descended from the MG1655 (K-12) *E. coli* strain. The PHL628 cell line has a point mutation at nucleic acid position 234 in the *ompR* gene, which is denoted as *ompR234*, creating a constitutively active OmpR protein (Vidal et al., 1998; Prigent-Combaret et al., 1999; Prigent-Combaret et al., 2001). OmpR is a transcription factor that binds to the promoter region of the *csgD* gene, the resultant protein product of which, CsgD, is a transcription factor that increases the expression of the *csgBA* operon, which, in turn, increases the amount of curli and enhances biofilm formation and growth. Curli are quaternary protein structures associated with robust biofilm formation in *E. coli*, and have been shown to aid in initial adhesion of the bacterial cells to a surface (Wong et al., 2017). Temperatures below 30 °C are conducive to high levels of curli production, and therefore also biofilm production, in most wild-type (WT) strains (Barnhart and Chapman, 2006). Interestingly, a flagellum-deficient derivative of this strain has been constructed (via knockout of the flagellin gene *fliC*, PHL1039) and, when compared to PHL628 (motile cells) grown in minimal medium at 30 °C, there was no advantage observed in either initial surface colonization or biofilm maturation with motility: “The strong presence of curli seems to override the need for flagella in biofilm formation of ompR234 strains” (Prigent-Combaret et al., 2000). Characterization of the biofilms formed by PHL628 cells has revealed that 142 genes are upregulated in sessile PHL628 cells as compared to planktonic cells; ten of these upregulated genes are involved in energy metabolism and 34 genes encode transport and/or binding proteins (Junker et al., 2006; Junker et al., 2007). We are using this strain because it effectively replicates the robust biofilm growth seen in pathogenic clinical strains without the human health hazards afforded by working with those strains.

We have overexpressed the transcription factor FliA in the PHL628 *E. coli* strain at varying temperatures in order to understand the nuanced dynamics of how a constitutively active ompR transcription factor and an exogenously overexpressed FliA transcription factor simultaneously influence biofilm formation and composition. We explore the role of the extracellular structures like curli and flagella on biofilm formation, as well as the protein and carbohydrate composition of the EPS using biochemical assays, immunoblotting and confocal laser scanning microscopy (CLSM).

## 2 Results

**Figure 1:**
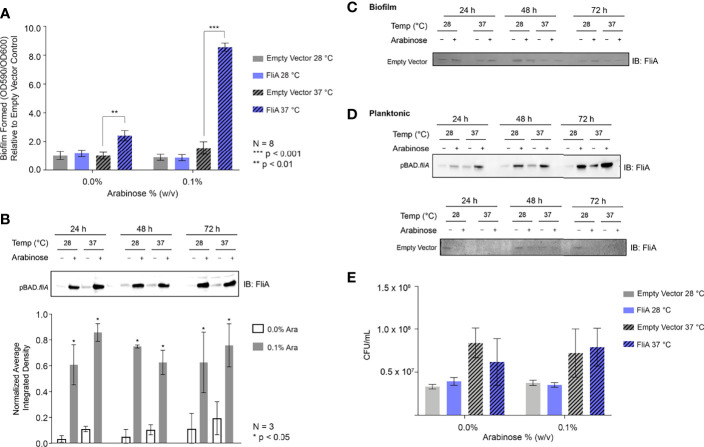
Crystal violet biofilm growth assay and FliA overexpression. **(A)** Amount of biofilm grown after 48 h in 96-well polystyrene plates. Significance is reported as standard deviation and results were analyzed by a two-way ANOVA. A representative anti-FliA immunoblot of **(B)** biofilm pBAD.fliA PHL628 cells over the course of 72 h. Graph x-axis corresponds with gel lane directly above bar. Quantitation of pixel density completed in ImageJ and significance is reported as standard deviation and analyzed by an unpaired Student’s t-test. Representative anti-Flia immunoblots of **(C)** biofilm empty vector PHL628 cells and **(D)** planktonic pBAD.fliA and empty vector PHL628 cells over the course of 72 h. **(E)** Colony counting experiment after 48 h growth on ampicillin-containing agar plates (N = 4).

We generated inducible overexpression plasmids of the transcription factor FliA (rpoF, sigma28) to better understand FliA’s influence on biofilm production and composition at varying temperatures. Using the PHL628 strain of *E. coli*, a K-12 strain that has a mutation in the OmpR protein that promotes the production of CsgA, a major component of extracellular curli, we first investigated the impact of FliA overexpression on biofilm growth using a well-established crystal violet microtiter plate assay (Merritt et al., 2005). After either 24 or 48 h, we measured the growth of biofilm with or without overexpression of FliA, as induced by the addition of arabinose (24 h: **Supplemental Figure S1A**, 48 h: **Figure 1A**). We observed a significant increase in biofilm after 48 h when FliA was overexpressed at 37°C.

We examined whether this large increase in biofilm production with FliA overexpression could be surface dependent. We grew biofilm for the microtiter plate assay in polystyrene 96-well plates. Several of the assays we use to qualitatively and quantitatively examine extracellular structures and biofilm matrix rely on confocal laser scanning microscopy (CLSM), which utilizes uncoated soda-lime glass slides. We repeated the above experiment, varying the temperature and overexpression of FliA, using these glass slides as a substrate and found that growth was more irregular than in the 96 well plate. While we did observe the increased growth at 37°C with FliA overexpression we did not observe this trend to the same magnitude as we did in polystyrene plates (48 h: **Figure S1B**). We compared the amount of biofilm grown on these slides with the same slide type pre-treated with poly-L-lysine, a polymeric coating that is often used to enhance cell adhesion to different substrates. We did not observe a notable change in amount of biofilm formed between coated and uncoated slides (**Figure S1C**), therefore, we continued our experiments with uncoated slides.

We confirmed overexpression and subsequent transcription factor activity of FliA at the mRNA transcript level using quantitative PCR (**Figure S2**). We monitored the transcript levels of *fliA*, *yhjH*, a phosphodiesterase, and *tar*, which encodes a chemotaxis protein. The FliA transcription factor binds to the promoter region of both *yhjH* and *tar* to form the RNA polymerase holoenzyme and initiate transcription (Fitzgerald et al., 2014). We monitored the transcript level 10 min after induction of *fliA* overexpression with arabinose because the half-life of *E. coli* mRNA, the biomolecule quantified in this type of qPCR experiment, is usually extremely short (on the order of minutes) (Esquerré et al., 2015) and there are several endogenous negative feedback loops that work to mitigate *fliA* overexpression (Fitzgerald et al., 2014). Subsequently, we monitored the protein concentration of FliA over the course of 72 h under our experimental conditions by immunoblot (**Figure 1B**). Here we observed a marked increase in FliA with the addition of arabinose at all temperatures and time points. Endogenous protein levels of FliA in the non-induced pBAD.*fliA* cells are similar to those observed in the empty vector control cells (**Figure 1C**), which are low, as expected (Li et al., 2014). For planktonic cells we observed a similar pattern of induction for FliA overexpressing and control cells (**Figure 1D**). The changes we observe in amount of biofilm formed upon FliA overexpression were not due to changes in cell viability as confirmed by colony counting experiments (**Figure 1E**) and an oxidoreductase activity-based fluorescence assay (**Figure S1D**).

**Figure 2:**
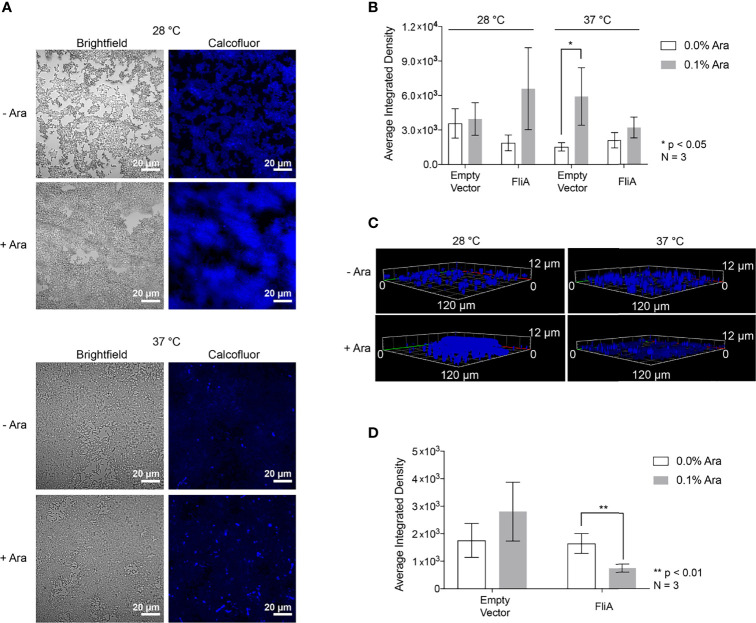
Calcofluor White CLSM. **(A)** Representative images of FliA-overexpressing cells after 48 h growth. White scale bar = 20 µm and Ara = arabinose. **(B)** Quantitation of average integrated density. **(C)** Z-stack images of representative stained fliA-overexpressing biofilms after 6 d growth rendered in Zeiss Zen Blue. **(D)** Quantitation of integrated density for biofilms grown at 28°C after 6 days. Significance is reported as standard deviation and analyzed by an unpaired two-tailed Student’s t-test.

The first extracellular biomolecules we examined were polysaccharides. We monitored the change in carbohydrate concentration in the EPS by both microscopy and biochemical assay. We used the fluorescent dye Calcofluor White (Fluorescent Brightener 28) to measure the amount of 1→3 and 1→4 β-linked polysaccharides in our biofilm samples. For biofilm growth on glass slides, we obtained the most reproducible results with biofilms grown on slides for 48 h or longer. Therefore, for CLSM experiments, we focused on 48 h and longer timepoints for analysis. For the longest timepoints (e.g., 6 days), we achieved the most consistent and reproducible growth of biofilm at the lower growth temperature (28°C). We stained biofilm grown on glass slides for either 48 h at both 28°C and 37°C or 6 days at 28°C with and without FliA overexpression and monitored fluorescence using CLSM (48 h: **Figures 2A, B**; **S3A**; 6 d: **Figures 2C**, **S3B**).

We quantified calcofluor fluorescence and found a marginally increased concentration of 1→3 and 1→4 β-linked polysaccharides in the EPS in all cases where arabinose had been added at higher temperatures after 48 h growth (**Figure 2B**). We collected three-dimensional z-stacks of the calcofluor-stained samples after 6 days and saw a significant decrease in the overall amount of polysaccharide when FliA was overexpressed at 28°C (**Figures 2C, D**). We quantified the amount of biomass and average biomass thickness for all 48 h and 6-day calcofluor-stained biofilms using the COMSTAT2 program in ImageJ (**Supplementary Table S3**). The COMSTAT analysis did not determine any significant difference in amount of Calcofluor-stained biofilm for our differing conditions, though it did follow the trend in reduced average biomass after 6 days of growth with FliA overexpression. We also measured and quantified the calcofluor fluorescence of biofilms grown on agar plates and observed no change in carbohydrate concentration at any experimental condition relative to empty vector control, though we do observe increased fluorescence overall at higher growth temperature (**Figure S4**). In order to obtain a more general quantification of carbohydrate content of the EPS, we used a colorimetric phenol sulfuric acid biochemical assay (DuBois et al., 1956). We found no significant increase in total carbohydrate concentration for biofilms overexpressing FliA after 48 h of growth at either growth temperature (**Figure S3C**).

The next biomolecule we investigated for its influence on biofilm formation in our system was the extracellular structural protein curli. As discussed previously, curli are highly expressed in the PHL628 strain of *E. coli* and facilitate adhesion to human cells and abiotic surfaces alike (Vidal et al., 1998; Beloin et al., 2008). Curli are subject to regulation, including temperature regulation, as they are most highly expressed below 30°C in most laboratory strains of bacteria, but are expressed in some clinical strains at 37°C (Barnhart and Chapman, 2006).

We quantified curli by biochemical assay using the azo dye Congo red, which has been used both in imaging of amyloid protein and in its quantification of amyloid by depletion assays (Reichhardt et al., 2015). Here, we quantified the amount of curli, an amyloid protein, observed at varying timepoints and temperatures for *E. coli* PHL628 cell in their planktonic and biofilm states with or without overexpression of the FliA transcription factor. A greater percentage depletion of Congo red from the supernatant fraction suggests a higher concentration of curli amyloid on the incubated cells. We grew biofilm on agar plates and harvested the EPS by gentle washing and centrifugation to separate the cellular fraction as described previously (Chiba et al., 2015). We incubated whole cells from biofilm and planktonic cultures with Congo red dye and measured the resultant depletion of dye from the supernatant fraction by UV-visible absorbance.

**Figure 3:**
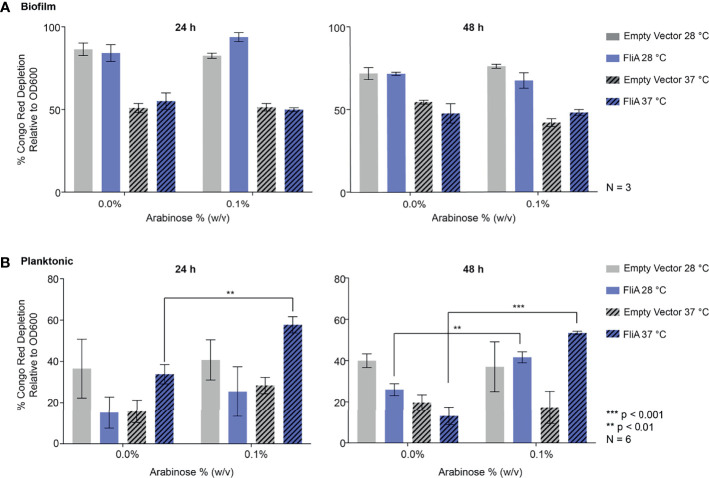
Quantitation of curli in PHL628 empty vector and FliA-overexpressing cells. Congo red depletion assay for **(A)** biofilms and **(B)** planktonic cells grown for 24 and 48 h (left, right, respectively). Significance is reported as standard deviation and analyzed by an unpaired two-tailed Student’s t-test.

For biofilm cells, we observed no differences in Congo red absorption with FliA overexpression relative to empty vector at either temperature studied (**Figure 3A**). We did observe a notable decrease in Congo red absorption at higher temperatures compared to lower growth temperatures, which we expected, as curli preferentially form at lower temperatures. Interestingly, for planktonic cells, we observed that with overexpression of FliA at both 28°C and 37°C, there is a significant increase in Congo red depletion, suggesting increased curli concentration compared to the control at both 24 h and 48 h (**Figure 3B**). As expected, all planktonic cells had lower concentrations of curli, as evidenced by less Congo red depletion, compared to biofilm cells.

**Figure 4:**
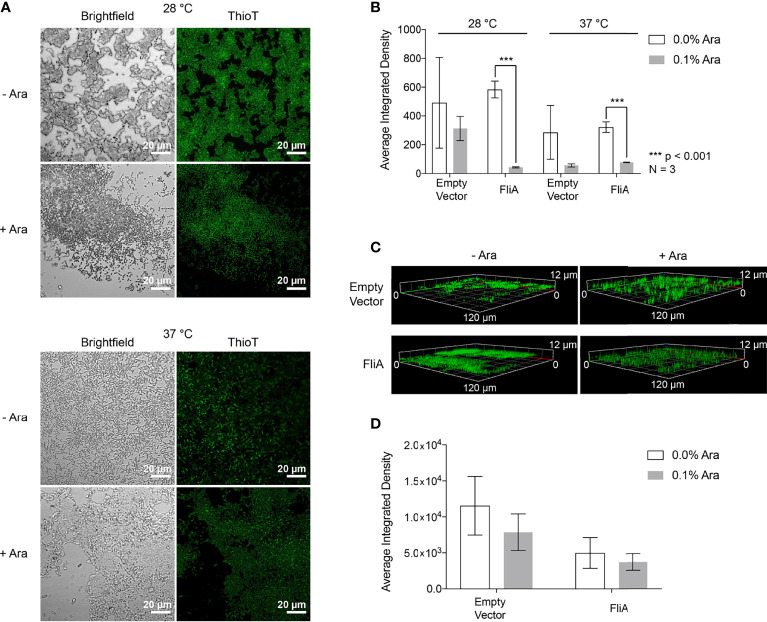
CLSM Analysis of thioflavin T-stained biofilms. **(A)** Representative images of pBAD.*fliA* PHL628 cells under differing experimental conditions after 48 h growth. Ara, arabinose. **(B)** Quantitation of integrated density of samples at 28°C and 37°C after 48 h growth. **(C)** Z-stack images of representative 6-day thioflavin T-stained biofilms rendered in Zeiss Zen Blue. **(D)** Quantitation of the average integrated density of 6-day biofilms. Significance is reported as a two-tailed Student’s t-test.

We sought to examine the role of curli in biofilm formation and composition separately from the Congo red assay. The fluorescent dye thioflavin T (4-(3,6-dimethyl-1,3-benzothiazol-3-ium-2-yl)-N,N-dimethylaniline chloride) binds to amyloid proteins like curli through interactions with cross-β sheet structures, and has been used for amyloid quantification in laboratory and clinical samples for decades (Schlafer and Meyer, 2016). We used confocal laser scanning microscopy (CLSM) to visualize the binding of thioflavin T to biofilms grown on glass slides under our experimental conditions of FliA overexpression at varying temperatures and times of growth (pBAD.*fliA* cells: **Figure 4A**, empty vector cells: **Figure S5**).

After 48 h, we observed differences in localization of curli and more dispersed, weaker fluorescence throughout the biofilm at both 28 °C and 37 °C with FliA overexpression (**Figure 4A**). We quantified fluorescence intensity of our CLSM samples by randomly selecting five identically sized regions for analysis and averaging their integrated density using the software ImageJ (**Figure 4B**). At both 28°C and 37°C, we observe a significant decrease in thioflavin T fluorescence with FliA overexpression, suggesting a decreased concentration of curli is present in those biofilm samples. In the empty vector control, we did not see a significant change in thioflavin T fluorescence under the same experimental conditions at 28°C, and a non-significant decrease in fluorescence at 37°C (**Figures 4B** and **S5**). We observed morphological differences in the biofilms between the 28°C and 37°C temperatures, with the biofilms grown at 28°C displaying more aggregative and clumping morphology and the biofilms grown at 37°C exhibiting more evenly dispersed cell coverage of the glass slides. We collected three-dimensional images of the thioflavin T-stained biofilms. For 6-day biofilms, we observed the presence of curli throughout the entire depth of the collected z-stack (12 µm), though the greatest fluorescence intensity was measured at a depth of 3.5 – 5.5 µm for all samples, or the top 1/3 – 1/2 of the biofilm for all samples analyzed (**Figure 4C**). When we averaged the integrated density of these 6-day biofilms at 28°C, we found that there was no longer a significant difference in amount of curli stained with FliA overexpression (**Figure 4D**).

We examined if the increased presence of curli at 37°C was also reflected in the concentration of the major structural subunit of curli, CsgA. We monitored the protein concentration of CsgA by immunoblot for both planktonic and biofilm cultures at varying times, temperatures and with or without overexpression of FliA (**Figure S6**). For all experimental conditions, we did not observe any notable changes in CsgA concentration in biofilm or planktonic cells by immunoblotting.

**Figure 5:**
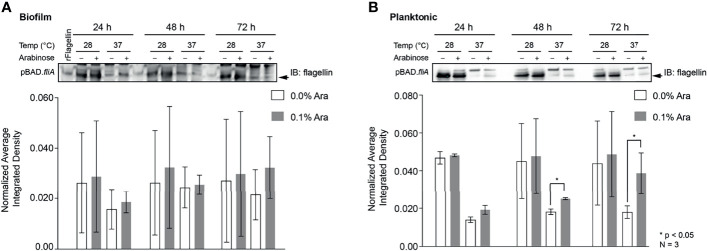
Quantitation of the protein flagellin in PHL628 cells with fliA overexpression. An anti-flagellin immunoblot of **(A)** biofilm and **(B)** planktonic pBAD.*fliA* PHL628 cells over the course of 72 h. Quantification of pixel density completed in ImageJ. Graph x-axis corresponds with gel lane directly above bar. Significance is reported as an unpaired Student’s t-test.

Other groups have reported that the overexpression of FliA in their strains of bacteria have resulted in the increased number of flagella per bacterium and enhanced motility (Shimada et al., 2017; Yang et al., 2019). In order to examine the influence of FliA overexpression on motility in PHL628 cells at varying temperatures, we quantified the total amount of flagellin (FliC) present by immunoblotting and saw markedly different quantities of flagellin present when cells were in biofilm versus the planktonic state (**Figures 5A, B** and **S7**). Interestingly, there was no significant difference in amount of flagellin present in biofilm samples with FliA overexpression for the duration of the experiment, but, for planktonic cells, we did observe significant increased concentration of flagellin at later time points. When bacteria were in their planktonic state, lower temperature growth resulted in significantly higher levels of flagellin for both FliA overexpressing and empty vector control cells.

**Figure 6:**
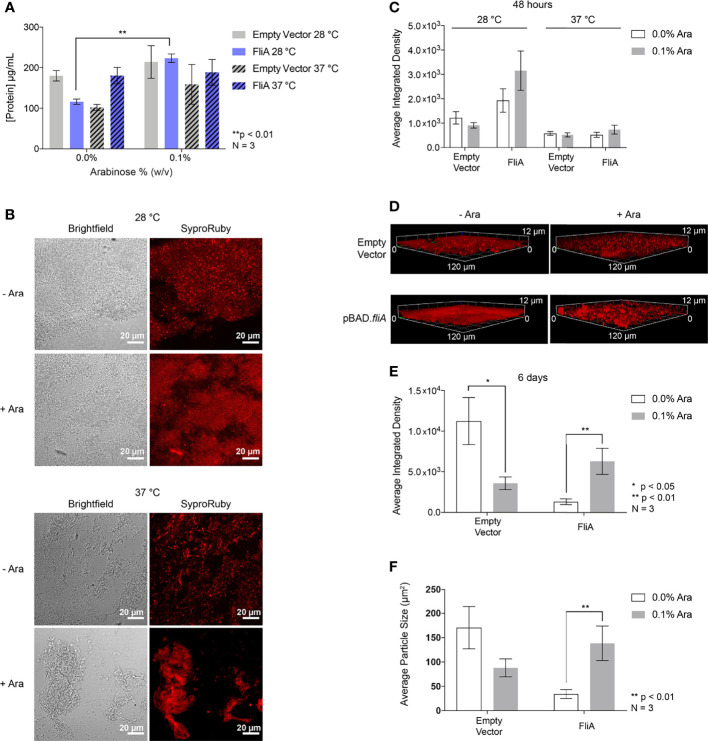
Total protein quantification and imaging. **(A)** Total protein concentration of the EPS from pBAD.*fliA* PHL628 biofilm measured by colorimetric BCA assay after 48 h. **(B)** Representative Sypro Ruby-stained CLSM images after 48 h growth and **(C)** quantitation of integrated density of biological triplicates. **(D)** Representative Sypro Ruby-stained CLSM z-stack images after 6 d growth at 28°C and **(E)** integrated density quantitation. **(F)** Average particle size determined by thresholding in ImageJ after 6 d growth at 28°C. Ara, arabinose. Significance is reported as an unpaired two-tailed Student’s t-test.

Finally, we sought to understand if any changes in overall protein concentration of the EPS could contribute to greater biofilm formation when FliA was overexpressed. We measured the protein concentration of EPS harvested from biofilms grown on agar plates for 48 h using a colorimetric assay and observed a significant increase in total protein concentration at 28°C with FliA overexpression (**Figure 6A**). We found that there was no significant change in the total protein concentration of the EPS at 37°C with FliA overexpression.

In order to observe any changes in distribution of protein in the EPS under our experimental conditions, we stained biofilm grown on glass slides with FilmTracer Sypro Ruby to image the morphology of the biofilm and quantify protein concentration by CLSM (**Figure 6B**). We observed no significant changes in amount of stained biofilm compared to control with any experimental condition after 48 h (**Figures 6C** and **S9**). We examined three-dimensional morphology and protein distribution after 6 days of growth (**Figure 6D**). After 6 days of growth at 28°C, the amount of EPS protein stained with the overexpression of FliA significantly increased in comparison to controls (**Figure 6E**). We wanted to determine if the size of the stained areas of the biofilm changed with either arabinose addition or FliA overexpression. By using the thresholding function in ImageJ and analyzing the size of “particles” or regions of fluorescence, we were able to quantify the difference in mean fluorescent area for our different conditions (**Figure 6F**). We observed that the mean fluorescent area increased significantly with the overexpression of FliA, indicating that there are larger, contiguous swaths of fluorescent stain, and, by proxy, biofilm, when FliA is overexpressed compared to controls.

Additionally, we used this total protein stain to quantify the overall biomass and thickness of our biofilms using the COMSTAT 2.1 software package for ImageJ (**Table 1**) (Heydorn et al., 2000; Vorregaard, 2008). We found that FliA overexpression increased the observed biomass at 48 hours for both temperatures significantly, and also increased the average thickness of the biofilm at 37°C. We did not observe any significant changes after 6 days of growth, though we do observe greater biomass for all experimental conditions.

**Table 1:** COMSTAT 2 measurements of Sypro Ruby-stained biofilms.

Time	Temperature	Plasmid	Arabinose % (w/v)	BioMass (µm^3^/µm^2^)	Average Thickness (µm)
48 h	28°C	Empty Vector	0.0	2.54 ± 0.95	10.27 ± 5.26
			0.1	2.95 ± 1.09	6.99 ± 3.09
		pBAD.*fliA*	0.0	6.91 ± 1.47	11.00 ± 1.41
			0.1	10.01 ± 1.53*	15.00 ± 1.42*
	37°C	Empty Vector	0.0	2.19 ± 0.98	5.22 ± 1.73
			0.1	4.10 ± 2.58	8.83 ± 3.70
		pBAD.*fliA*	0.0	4.85 ± 2.16	7.55 ± 3.88
			0.1	8.30 ± 1.39**	15.10 ± 0.58*
6 d	28°C	Empty Vector	0.0	14.56 ± 0.69	16.50 ± 0.71
			0.1	10.99 ± 4.77	15.23 ± 6.93
		pBAD.*fliA*	0.0	12.65 ± 2.50	15.67 ± 2.08
			0.1	10.65 ± 5.70	13.50 ± 2.12

Significance is reported as standard deviation and analyzed by unpaired two-tailed Student’s t-test. **p < 0.01, *p < 0.05, N = 3.

## 3 Discussion

The overexpression of FliA in PHL628 *E. coli* cells resulted in a significant increase in biofilm growth in polystyrene plates at higher temperatures as compared to empty vector controls (**Figure 1A**). We observed modest biofilm growth for pBAD.*fliA* PHL628 cells at higher temperatures even without the induction of FliA expression with arabinose, suggesting that the plasmid may be leaky. To determine if we were observing greater-than endogenous expression levels without arabinose induction, we examined the amount of *fliA* mRNA transcript (**Figure S2**) and protein (**Figures 1C** and **S1D**). While we do observe a modestly increased *fliA* expression level even without arabinose induction (**Figure S2**), the levels of active transcription factor are significantly different than non-induced plasmid after arabinose induction. We examined whether the type of substrate influences the effect of FliA overexpression on biofilm growth, as the type of substrate can dramatically change the ability of biofilm to form (Katsikogianni and Missirlis, 2004). We measured biofilm growth on glass slides that were either uncoated or coated with sterile poly-L-lysine. On glass slides, we found that the amount of biofilm formed with FliA overexpression increased significantly at higher temperatures, but to a more modest extent in comparison to the 96-well polystyrene-based assay (**Figure S1B**). We found that using poly-L-lysine coating on the silica slides resulted in no difference in the amount of biofilm grown (**Figure S1C**). Our optimal timing of biofilm growth at both low (28°C) and high (37°C) temperatures was 48 h. We were also able to observe robust biofilm growth at lower temperatures after 6 days. Additionally, the differences we observe in biofilm formation with FliA overexpression are not explained by differences in cell viability, as demonstrated with cell counting (**Figure 1E**) and a fluorescence-based viability assay (**Figure S1D**).

We first examined the concentration and distribution of carbohydrates in the EPS, as their presence can lead to more robust biofilm formation (Serra et al., 2013). Using CLSM, we stained biofilms with calcofluor in order to visualize 1→3 and 1→4 β-linked polysaccharides, the most abundant of which is typically cellulose in microbial cultures. Interestingly, K-12 strains of *E. coli* do not produce cellulose, despite having all of the necessary operons and regulatory genes for cellulose production (Zogaj et al., 2001; Da Re and Ghigo, 2006). Here, we see significant calcofluor fluorescence of biofilms grown both on glass microscopy slides and on agar plates (**Figure 2** and **Figure S3**), suggesting that there is an abundance of 1→3 and 1→4 β-linked carbohydrates in the EPS. After 48 h growth, we did not observe any significant changes in calcofluor fluorescence with FliA overexpression. Interestingly, we did observe an increase in calcofluor fluorescence in our empty vector control with the addition of arabinose. This was not wholly unexpected, as we are using a pentose sugar and nutrient that could be independently changing the makeup of the EPS, though we do not observe this consistently with our samples overexpressing FliA. Notably, researchers recently discovered that the use of arabinose in *Salmonella* influences biofilm formation, specifically through its influence on intracellular cyclic-di-GMP levels (Vasicek et al., 2021). We did observe a decrease in calcofluor fluorescence at low temperature after 6 days of growth when FliA is overexpressed (**Figure 2D**). At present, the link between this decrease in EPS carbohydrate and FliA overexpression is unknown. When examining the total carbohydrate concentration of the EPS by phenol-sulfuric acid assay, we found no notable difference in carbohydrate concentration of the EPS at either 28°C or 37°C with FliA overexpression (**Figure S3C**). In total, these results suggest that there is no significant change in EPS carbohydrate that would lead to increased biofilm growth with FliA overexpression.

To better understand why the overexpression of FliA influences biofilm growth and maturation, especially at higher temperatures, we examined the prevalence of curli, an extracellular structure that plays a role in adhesion and biofilm maturation using biochemical and immunoblot assays (**Figure 2**) and CLSM (**Figures 3** and **S5**). Staining with thioflavin T for curli visualization by CLSM revealed a significant decrease in curli with FliA overexpression at both 28°C and 37°C after 48 h (**Figure 3B**). However, no significant change upon FliA overexpression was observed after 6 days of growth. When quantifying curli using alternative methods (Congo red depletion, **Figure 2A**, and anti-CsgA immunoblotting, **Figure S6**), we did not observe any significant differences when FliA was overexpressed in biofilm. This inconclusive result could be due to the different methods of growth. Biofilms grown on glass slides did not provide sufficient material for these assays, and therefore biofilms formed for biochemical and immunoblot assays were grown on agar plates. Separately, we could be observing the non-specific binding of thioflavin T or Congo red to other amyloid protein, or proteins containing large regions of β-strands in our complex sample, or even other biomolecules like polysaccharides, leading to conflicting results. (Namioka et al., 2020) Regardless, the lack of curli present in biofilms examined by CLSM does not likely explain the robust growth that we see at higher temperatures for PHL628 cells overexpressing FliA.

When probing biofilm and planktonic cultures overexpressing FliA for the protein concentration of flagellin, the primary protein that assembles into the flagellum structure, we observed dramatically increased concentrations of flagellin for all experimental conditions at 28°C compared to the higher temperature condition. The increased amount of flagellin at lower temperatures is not surprising: reduced motility at higher temperatures (e.g., 42°C), caused by a lack of flagella, has been observed for *E. coli* (Morrison and McCapra, 1961; Li et al., 1993). Additionally, in our immunoblots we see two, distinct bands: a lower band, which we quantified and which has a significantly greater intensity at lower temperatures, and a slightly higher molecular weight band with greater intensity at higher growth temperature. We did observe significant increases in flagellin concentration with FliA overexpression at 37°C at later timepoints for planktonic cells (**Figure 5B**), but not in biofilm cells (**Figure 5A**). Again, this was not surprising, as biofilm cells routinely lose their flagella during adhesion and maturation, and the differences that we observe in planktonic cells might be diminished in the biofilm (Sharma et al., 2016).

Our future studies will also include an examination of the anti-sigma factor FlgM, a small protein that binds to FliA, inhibiting FliA binding to DNA, and its role in creating negative feedback loops that might be tempering the overexpression response of FliA (Park et al., 2001; Barembruch and Hengge, 2007). FlgM expression is induced by FliA and is inhibited by CsgD, a transcriptional regulator that has its own transcription regulated by OmpR, a protein that is constitutively active in PHL628 cells (Dudin et al., 2014; Perni et al., 2016). This complex network of interactions could explain both the tempered nature of our CLSM and biochemical results, despite exogenous overexpression of FliA.

Finally, we sought to determine if the amount of protein in the EPS was leading to increased biofilm formation by measuring the total protein concentration by colorimetric assay (**Figure 6A**). While we observed a modest, yet significant increase in protein concentration at lower growth temperature with FliA overexpression, there was no difference in protein concentration at higher growth temperatures. We examined whether the protein distribution in the biofilm EPS changes with FliA overexpression by biochemical assay and staining biofilms with Sypro Ruby and measuring fluorescence by CLSM (**Figures 6A, B**). We note that harvesting the EPS and measuring total protein concentration by colorimetric assay may not be as sensitive to changes in overall protein concentration. While the method we use to separate cells from EPS is gentle, there is a possibility of contamination of the EPS sample with intracellular protein. Sypro Ruby is an extracellular protein stain and is also commonly used to examine overall biofilm morphology, and we collected data regarding average biomass and thickness for our different experimental conditions by analyzing our 3-dimensional biofilm images with COMSTAT 2 (**Table 1**) (Vorregaard, 2008). After 48 h, we did not see any significant differences in average EPS protein concentration at either temperature, though there was notably less protein in the EPS overall at the higher growth temperature (**Figure 6C**). After 6 days of growth at the lower temperature, we observed a significant increase in EPS protein with overexpression of FliA (**Figure 6E**). Additionally, the staining of the biofilm using Sypro Ruby revealed that there were larger regions of connected biofilm (quantified as particle size in ImageJ) with FliA overexpression (**Figure 6F**). COMSTAT results supported the significant differences in biomass with overexpression of FliA at both temperatures studied after 48 h (**Table 1**). We plan to continue our exploration of the role of protein in the EPS using quantitative liquid chromatography-mass spectrometry (LC-MS) to determine whether the concentration of specific EPS proteins are differentially changing with FliA overexpression, which could explain the changes in biofilm amount we have observed (Suryaletha et al., 2019). While we were unable to make direct comparison about biofilm growth and composition between the PHL628 strain and the K-12 MG1655 parent strain, which does not express a constitutively active OmpR, because we were not able to grow robust biofilms with the parent strain under our optimized experimental conditions, we plan to re-optimize growth conditions for this strain for EPS harvesting for LC-MS analysis.

## 4 Conclusion

We exogenously overexpressed the transcription factor FliA in PHL628 *E. coli* cells that robustly form biofilm due to a constitutively active OmpR transcription factor, which resulted in increased biofilm formation, especially at 37°C. We examined whether this increase in biofilm was due to changes in predominant biomolecules in the extracellular polymeric substance of the biofilm using CLSM and biochemical and immunoblot assays. We did not see any notable changes in either curli or carbohydrate amount in the EPS of biofilm samples. Still, we did observe significant changes in the amount of protein in the EPS by CLSM. Moreover, we found that FliA overexpression produced significantly more flagellin protein in planktonic samples, but did not result in significant differences in flagellin concentration in biofilm. Taken together, this suggests that one of the possible reasons for increased biofilm growth at higher temperatures is due to changes in protein concentration in the EPS. Our future experiments will focus on examining the identity and the changing concentration of the proteins in the EPS using quantitative LC-MS and carefully studying the complex regulatory network between the transcription factors FliA and OmpR and their downstream targets FlgM and CsgD using deletion studies, quantitative PCR, and immunoblotting.

## 5 Methods

### 5.1 Bacterial Growth and Strains

Ampicillin (IBI Scientific) or kanamycin (Sigma-Aldrich) antibiotics were used to select for the *E. coli* cells containing the desired exogenous plasmid. A 10% (w/v) arabinose stock solution was made by dissolving 1.00 g of L-(+)-Arabinose (Sigma-Aldrich) in 9.00 mL of ultrapure water and sterile filtering the solution. A 0.1% (w/v) crystal violet solution used to stain the microtiter plate biofilms was made by dissolving 0.0500 g of crystal violet powder (Sigma-Aldrich) in 50. mL of ultrapure water. The 30% acetic acid mixture used to solubilize the crystal violet was made by mixing 75 mL of acetic acid (Sigma-Aldrich) and 175 mL of ultrapure water.

All of the *E. coli* cell cultures were grown in Luria Broth (LB) composed of 20.00 g of LB Broth Lennox (Hardy Diagnostics) per liter of water. All the *E. coli* cell colonies were grown on LB plates composed of 25.00 g Lennox LB Broth (Hardy Diagnostics) and 15.00 g Agar Powder (Alfa Aesar) per liter of water. Depending on the experiment and type of selection needed, ampicillin, kanamycin and/or arabinose was added to the LB media and LB plates.

For the experiments involving FliA overexpression, chemically competent DH5α *E. coli* cells (New England BioLabs Inc.) and PHL628 *E. coli* cells (a kind gift from Anthony G. Hay, Ph.D., Cornell University) were used. Chemically competent PHL628 cells were prepared as previously described (Chung and Miller, 1993). The pBAD18 plasmid was a kind gift from Laura Romberg, Ph.D., Oberlin College (**Table 2**).

**Table 2:** *E. coli* strains and plasmids.

Strain or plasmid	Description	Reference or source
Strains		
PHL628	MG1655 malA-kan ompR234	Vidal et al., 1998
DH5α	LuxS supE44 ΔlacU169, (ϕ80 lacZΔM15) hsdR17, recA1, endA1, gyrA96, thi-1, relA1	Invitrogen
Plasmids		
pBAD18	Amp^R^ cloning plasmid containing arabinose inducible pBAD promoter	Guzman et al., 1995
pBAD18.fliA	pBAD18 harboring entire fliA gene from PHL628 downstream of the pBAD promoter	This study

The *fliA* gene was cloned behind an arabinose-controlled promoter in the pBAD18 plasmid using a restriction digest-based approach. The *fliA* gene was first amplified using polymerase chain reaction (PCR) from genomic PHL628 *E. coli* DNA with the Phusion polymerase (New England BioLabs Inc.) using a forward primer containing a NheI enzyme cut-site, and a reverse primer containing a SalI enzyme cut-site (**Supplemental Table S1**). The amplified *fliA* gene and a purified pBAD18 expression plasmid were digested with NheI (New England BioLabs Inc.) and SalI (New England BioLabs Inc.) enzymes at 37°C for 1 h to create sticky ends. The sequences of the pBAD18 plasmid and *fliA* gene were confirmed using a 1% agarose gel and they were both subsequently gel purified using the QIAquick Gel Extraction Kit and protocol (Qiagen), and the concentration of DNA was measured using a Nanodrop (ThermoScientific). Next, the digested pBAD18 plasmid and *fliA* gene were ligated together using a T4 DNA ligase (New England BioLabs Inc.) reaction with 37.5 ng of *fliA* DNA and 50 ng of pBAD18 DNA. The ligation reaction was incubated at room temperature for 20 min.

Following the ligation reaction, the pBAD18 plasmid with the *fliA* gene was transformed into chemically competent DH5α *E. coli* cells (Invitrogen) (Guzman et al., 1995). The pBAD18.*fliA* DNA construct was purified out of the DH5α *E. coli* cells using the QIAprep^®^ Spin Miniprep Kit (Qiagen). Upon confirmation by sequencing that the ligation reaction was successful, the pBAD18.*fliA* DNA construct was transformed into chemically competent PHL628 *E. coli* cells. All sequencing was performed at the Genomics Core Center in the Lerner Research Center at the Cleveland Clinic, Cleveland, Ohio.

Viability of bacteria with and without addition of arabinose were confirmed by colony counting experiments and a fluorescence-based assay. Colony counting experiments were conducted as follows: bacteria were grown overnight at 37°C or 28°C, and diluted 1:50. They were then allowed to reach an OD_600_ of 0.4 before they were serially diluted for spread plating from 3.2 x 10^8^ colony forming units (CFU) per 1 mL of growth medium to 5 x 10^-6^ CFU/mL. Bacteria grew for 48 h at 37°C or 28°C, after which colonies were counted by eye. We also measured cell viability using a resazurin-based fluorescence assay (alamarBlue, excitation 560 nm/emission 590 nm, Invitrogen). Briefly, bacteria were grown overnight at 37°C or 28 °C, diluted 1:50, and allowed to grow to OD_600_ 0.5. Cells were diluted to 10^8^ cells/mL with LB growth medium, and 180 µL of cells were added in triplicate to an opaque black 96-well plate. 20 µL of alamarBlue was added to each well and cells were incubated at 37°C for 4 hours. Fluorescence was measured using a Molecular Devices Spectramax M5 plate reader.

### 5.2 Biofilm Growth Experiments

Before each biofilm experiment, PCR and DNA visualization *via* a 1% agarose gel were used to confirm that the PHL628 *E. coli* cells contained either pBAD.*fliA* or empty vector. Starter cultures of PHL628 cells containing the pBAD18.*fliA* construct or PHL628 cells containing the empty pBAD18 plasmid (empty vector) were grown overnight at 37°C in LB with ampicillin in a shaking incubator at 190 rpm. After overnight growth, the OD_600_ of the cells was measured and cells were diluted to the same stock concentration of 3 x 10^7^ cells/mL with 0%, 0.05%, and 0.1% (w/v) arabinose. Two hundred microliters of each solution and 200 μL of LB with ampicillin were added to wells of UV-sterilized 96-well microtiter plates. Wells in the center of the microtiter plate were used to prevent the cultures from drying out. Microtiter plates were incubated at either 28°C or 37°C with UV-sterilized caps for 24 h or 48 h before they were analyzed.

After each biofilm microtiter plate was incubated for the designated amount of time, its OD_600_ was measured using a Bio-Rad Benchmark Plus Microplate Spectrophotometer. The bacterial cultures in each well were then discarded and the entire microtiter plate was rinsed several times in deionized water. Next, 300 µL of 0.1% (w/v) crystal violet stain was added to each well and the plate was left at room temperature for 10 min. After 10 min the crystal violet was removed from the plate and rinsed again before being allowed to dry at room temperature overnight. Once the stained microtiter plates were dry, the crystal violet adhered to the sides of each well was solubilized in 30% acetic acid. Three hundred microliters of 30% acetic acid was added to each well and the plate was left at room temperature for 15 min. The solution in each well was mixed by pipetting and 200 µL was transferred to a new microtiter plate. The OD_590_ was measured using a Bio-Rad Benchmark Plus Microplate Spectrophotometer. The OD_590_/OD_600_ ratio was used to quantify biofilm growth.

All biofilm samples for microscopy were grown on glass slides (Corning, 24 mm x 60 mm) immobilized in 50 mL conical tubes with 30 mL of LB with 100 µg/mL ampicillin and either 0.0% or 0.1% arabinose in shaking incubators at 190 rpm at the temperatures and times indicated. Each day, the level of LB was monitored and cultures were fed additional LB (+ampicillin, +/-arabinose) as needed. Slides were removed from the conical tubes, rinsed 3 x for 1 min with deionized water and stained as indicated below. Quantitative biofilm imaging was obtained by collecting data within 20 – 40 mm of the top of the slide and confirming the thickness of the biofilm by z-stack. Slides that did not grow sufficient biofilm or had the biofilm slough off during the staining process were not included in any analyses.

### 5.3 Quantitative RT-PCR

The relative mRNA expression levels of *fliA* and target genes were measured using qPCR. Bacterial cultures were grown overnight at 28°C or 37°C in a shaking incubator at 190 rpm. Cultures were diluted 0.0625:1 and were grown for 40 – 45 min, until they reached mid-log phase (OD_600_ = 0.5). After induction with arabinose for 10 min, the number of cells was determined by measuring the OD_600_. Approximately 2 x 10^8^ cells/mL were harvested by centrifugation for 8 min at 1300 x g.

RNA was extracted using the RNeasy Mini Kit (Qiagen) supplemented with RNeasy Protect Bacteria Mini Kit (Qiagen). qPCR reactions were performed on cDNA prepared from total cellular RNA using the QuantiTect Reverse Transcription Kit (Qiagen). The Rotor-Gene SYBR Green dye (Qiagen), cDNA, and appropriate primers purchased from Integrated DNA Technologies (**Supplemental Table S2**) were used for amplifications (45 cycles of 2 min at 95°C, 10 s at 95°C, 30 sec at 60°C) in a Qiagen Rotor-Gene Q instrument. All transcripts were normalized to the housekeeping gene *rrsA* and all measurements were performed in triplicate. Data was analyzed using the ΔΔCt method and error is reported as SEM.

### 5.4 Preparation of Biofilm and EPS Samples for Analysis

Biofilms grown for analysis of extracellular polymeric substances or immunoblotting were grown statically on 10 cm LB-agar plates supplemented with ampicillin (for selection of the pBAD-plasmid containing cells) and with or without 0.1% (w/v) arabinose. Biofilm cells and EPS were harvested as previously described (Chiba et al., 2015). Briefly, a 4 h starter culture was prepared, an OD_600_ of between 0.4 – 1.0 was reached, and 12.5 µL of culture was spotted onto LB-agar plates. The cultures were permitted to dry near a flame, then incubated for the times and temperatures indicated. Biofilms were then scraped off the surface of the agar using a sterilize cell scraper and resuspended in 1 mL of 1.5 M NaCl. Cells were centrifuged at 5000 x g for 10 min at room temperature. The supernatant was saved and analyzed as the EPS, while the cells were separately lysed and processed for immunoblotting.

### 5.5 Confocal Laser Scanning Microscopy

All stained slide samples were protected from exposure to natural and fluorescent light. Slides were incubated with thioflavin T (15 µM in 150 mM sodium phosphate buffer, pH 7.0, Sigma-Aldrich) in a covered 10 cm petri dish for 1 h at room temperature, then removed from the stain and dried overnight in a 28°C or 37°C incubator. A coverslip was attached and sealed with clear nail polish. Slides were incubated with 0.025% Calcofluor White (Sigma-Aldrich) in water for 1 min in a covered 10 cm petri dish at room temperature, then removed from the stain and dried overnight in a 28°C or 37°C incubator. A coverslip was attached and sealed with clear nail polish. FilmTracer Sypro Ruby biofilm matrix stain (Invitrogen) was added to the biofilm on the glass slide and incubated at room temperature for 30 min. The slide was rinsed, a coverslip was placed on the slide and it was immediately imaged. For those slides coated with poly-L-lysine (0.1% w/v solution in deionized water, Sigma-Aldrich), we first cleaned slides with an acidic alcohol solution (1% HCl in ethanol) and then, using a slide rack, immersed slides in a 1:10 diluted poly-L-lysine solution and incubated at room temperature for 5 minutes. Slides were then drained and dried in a sterile container overnight at room temperature before use.

Confocal laser scanning microscopy (CLSM) was employed to analyze the PHL628 biofilm cells and extracellular polymeric substances using various dyes on a Zeiss LSM 880 inverted confocal microscope (Carl Zeiss, Jena, Germany) equipped with MA-PMT and GaAsP array detectors. Samples were viewed through a 63X oil-immersion objective. Thioflavin T and FilmTracer Sypro Ruby stained samples were imaged by excitation from a 458 nm Argon laser. Calcofluor stained samples were imaged by excitation from a 405 nm diode array.

Fluorescence intensity analysis was completed by imaging five randomly located, identically sized areas on a slide in the same z-plane of greatest fluorescent intensity. Images were analyzed in ImageJ by determining a common threshold of fluorescence using Otsu thresholding and subsequently determining the mean integrated density of fluorescent areas using the “analyze particles” function (Schindelin et al., 2012; Schneider et al., 2012; Shihan et al., 2021). This analysis provided us with comprehensive information about the spread and size of stained biofilm in two dimensions, as well as fluorescence intensity. Z-stack images were visualized using Zeiss ZenBlue 2.3 software. Biomass and average thickness measurements of z-stacks were calculated using the COMSTAT 2.1 software (Heydorn et al., 2000; Vorregaard, 2008).

### 5.6 EPS Protein and Carbohydrate Assays

#### BCA Protein Assay

Protein concentration of the EPS was determined using the Pierce bicinchoninic acid (BCA) protein assay kit (Thermo Scientific). 200 µL of treated samples and the treated BSA standards were pipetted into clear 96-well plates (Corning) and the absorbance at 530 nm was measured on a Spectramax M5 plate reader (Molecular Devices).

#### 5.6.2 Phenol Sulfuric Acid Carbohydrate Assay

EPS samples (200 µL) were added to 200 µL 5% (v/v) phenol (Sigma Aldrich) and 1.0 mL concentrated sulfuric acid (Flinn Scientific) in individual 15 mL conical tubes, mixed and incubated at room temperature for 10 minutes. Samples were mixed well and placed in a 30°C water bath for 20 minutes. Glucose (Alfa Aesar) standards were prepared simultaneously. 200 µL of the treated samples were pipetted into a clear 96-well plates (Corning) and the absorbance at 490 nm was measured on a Spectramax M5 plate reader (Molecular Devices).

#### 5.6.3 Calcofluor White Plates

Overnight cultures of bacteria were diluted to OD_600_ of 0.5, then 12 µL were plated on LB-agar plates. Bacteria were grown statically at either 28 or 37°C on LB-agar plates with calcofluor (0.05 mg/ml) and ampicillin (100µg/mL), with or without 0.1% (w/v) arabinose. (Adamus-Białek et al., 2019) The level of calcofluor bound up with cellulose was measured on a ChemiDoc MP imager (Bio-Rad) with a Blue Epi Illumination excitation source and a 523/38 filter. Relative cellulose production was determined based on the ratio between the fluorescence intensity [integrated density, after background subtraction by rolling ball algorithm (250 pixels)] to area of the colony, as measured in ImageJ. This method of calculation was used to obtain relative cellulose production independent of bacterial growth.

#### 5.6.4 Congo Red Depletion Assay

Congo red depletion assays were carried out as previously described (Reichhardt et al., 2015). Biofilm samples were grown on agar plates (with 100 µg/mL ampicillin and 0.1% arabinose, as indicated) and harvested by sterile scraping as described above. Planktonic cells were harvested from cultures growing in the logarithmic growth phase, and cells were centrifuged at 5000 x g for 10 min to remove old growth medium. All cells were resuspended in 1 mL of fresh LB broth and OD600 was used to create an absorbance dilution series between 0.2 and 1.0 OD600. Optimum cell concentration was determined as an absorbance of 0.8 at OD600. A sterile filtered Congo red (Sigma Aldrich) stock solution prepared in LB (1 mg/mL), was added to samples to a final Congo red concentration of 10 µg/mL. Samples were incubated at 4°C for 24 hr with rocking. Cells were pelleted at 10,000 x g for 5 min. The supernatant was transferred to a clear 96-well polystyrene plate and the absorbance at 500 nm was read on a Spectramax M5 plate reader (Molecular Devices). Error is reported as the standard deviation of at least three biological replicates.

### 5.7 Immunoblotting

Cells were lysed in 50 mM Tris buffer, pH 7.5 containing 0.1% TritonX (Fisher Scientific) and supplemented with protease inhibitor mixture (Roche). Protein lysate concentrations were normalized by Bradford assays (Bio-Rad). Lysates or media were boiled for 10 min in Laemmli buffer + 100 mM DTT before loading onto either 15% or 20% SDS-PAGE gels (Stain-Free TGX, Bio-Rad). Stain-Free analysis of gel slabs was conducted on a GelDock EZ system (Bio-Rad). Proteins were transferred from gel slabs to nitrocellulose membrane.

Blots were probed with the following primary antibodies: mouse monoclonal anti-FliA (1:500, Biolegend 1RF18), polyclonal rabbit anti-CsgA (1:10,000, a kind gift from Matthew Chapman, Ph.D., University of Michigan), rabbit polyclonal anti-flagellin (1:1,000, Abcam ab93713). Secondary antibodies conjugated to HRP were used, and blots were developed with an ECL detection kit (Amersham Bioscience). Chemiluminescence was visualized using a ChemiDoc MP system (Bio-Rad). Densitometry was quantified using ImageJ, and bands were normalized to total lane intensity determined by Stain-Free imaging (Gassmann et al., 2009; Taylor and Posch, 2014).

## Data Availability Statement

The original contributions presented in the study are included in the article/**Supplementary Material**. Further inquiries can be directed to the corresponding author.

## Author Contributions

LR conceived the original project idea. LR, SU, LB, and MP planned the experiments. All data was collected by LB, MP, KN, EB, JH, and SU and analyzed in collaboration with LR. The manuscript was prepared by LR, with all authors providing critical feedback during the data collection, analysis and writing process. All authors contributed to the article and approved the submitted version.

## Funding

We thank the Research Corporation Cottrell Scholar Award, the NSF (MRI, CHE-1828041), and Oberlin College for financial support. LB was supported by the Robert Rich fund (Oberlin College).

## Conflict of Interest

The authors declare that the research was conducted in the absence of any commercial or financial relationships that could be construed as a potential conflict of interest.

## Publisher’s Note

All claims expressed in this article are solely those of the authors and do not necessarily represent those of their affiliated organizations, or those of the publisher, the editors and the reviewers. Any product that may be evaluated in this article, or claim that may be made by its manufacturer, is not guaranteed or endorsed by the publisher.

## References

[B1] Adamus-Białek W. Vollmerhausen T. L. Janik K. (2019). Hydrogen Peroxide Stimulates Uropathogenic Escherichia Coli Strains to Cellulose Production. Microb. Pathog. 126, 287–291. doi: 10.1016/j.micpath.2018.11.020 30447422

[B2] Barembruch C. Hengge R. (2007). Cellular Levels and Activity of the Flagellar Sigma Factor FliA of Escherichia Coli Are Controlled by FlgM-Modulated Proteolysis. Mol. Microbiol. 65, 76–89. doi: 10.1111/j.1365-2958.2007.05770.x 17537210

[B3] Barnhart M. M. Chapman M. R. (2006). Curli Biogenesis and Function. Annu. Rev. Microbiol. 60, 131–147. doi: 10.1146/annurev.micro.60.080805.142106 16704339 PMC2838481

[B4] Barrios A. F. G. Zuo R. Ren D. Wood T. K. (2006). Hha, YbaJ, and OmpA Regulate Escherichia Coli K12 Biofilm Formation and Conjugation Plasmids Abolish Motility. Biotechnol. Bioeng. 93, 188–200. doi: 10.1002/bit.20681 16317765

[B5] Belas R. (2014). Biofilms, Flagella, and Mechanosensing of Surfaces by Bacteria. Trends Microbiol. 22, 517–527. doi: 10.1016/j.tim.2014.05.002 24894628

[B6] Beloin C. Roux A. Ghigo J. M. (2008). “Escherichia Coli Biofilms,” in Bacterial Biofilms Current Topics in Microbiology and Immunology (Berlin, Heidelberg: Springer, Berlin, Heidelberg), 249–289. doi: 10.1007/978-3-540-75418-3_12 PMC2864707 18453280

[B7] Chaban B. Hughes H. V. Beeby M. (2015). The Flagellum in Bacterial Pathogens: For Motility and a Whole Lot More. Semin. Cell Dev. Biol. 46, 91–103. doi: 10.1016/j.semcdb.2015.10.032 26541483

[B8] Chiba A. Sugimoto S. Sato F. Hori S. Mizunoe Y. (2015). A Refined Technique for Extraction of Extracellular Matrices From Bacterial Biofilms and Its Applicability. Microb. Biotechnol. 8, 392–403. doi: 10.1111/1751-7915.12155 25154775 PMC4408173

[B9] Chung C. T. Miller R. H. (1993). Preparation and Storage of Competent Escherichia Coli Cells. Meth. Enzymol. 218, 621–627. doi: 10.1016/0076-6879(93)18045-e 8510550

[B10] Claret L. Miquel S. Vieille N. Ryjenkov D. A. Gomelsky M. Darfeuille-Michaud A. (2007). The Flagellar Sigma Factor FliA Regulates Adhesion and Invasion of Crohn Disease-Associated Escherichia Coli *via* a Cyclic Dimeric GMP-Dependent Pathway. J. Biol. Chem. 282, 33275–33283. doi: 10.1074/jbc.M702800200 17827157

[B11] Costerton J. W. Stewart P. S. Greenberg E. P. (1999). Bacterial Biofilms: A Common Cause of Persistent Infections. Science 284, 1318–1322. doi: 10.1126/science.284.5418.1318 10334980

[B12] Da Re S. Ghigo J.-M. (2006). A CsgD-Independent Pathway for Cellulose Production and Biofilm Formation in Escherichia Coli. J. Bacteriol. 188, 3073–3087. doi: 10.1128/JB.188.8.3073-3087.2006 16585767 PMC1447019

[B13] Donlan R. M. Costerton J. W. (2002). Biofilms: Survival Mechanisms of Clinically Relevant Microorganisms. Clin. Microbiol. Rev. 15, 167–193. doi: 10.1128/cmr.15.2.167-193.2002 11932229 PMC118068

[B14] DuBois M. Gilles K. A. Hamilton J. K. Rebers P. A. (1956). Colorimetric Method for Determination of Sugars and Related Substances. Anal. Chem. 28, 3, 350–3, 356. doi: 10.1021/ac60111a017

[B15] Dudin O. Geiselmann J. Ogasawara H. Ishihama A. Lacour S. (2014). Repression of Flagellar Genes in Exponential Phase by CsgD and CpxR, Two Crucial Modulators of Escherichia Coli Biofilm Formation. J. Bacteriol. 196, 707–715. doi: 10.1128/JB.00938-13 24272779 PMC3911157

[B16] Esquerré T. Moisan A. Chiapello H. Arike L. Vilu R. Gaspin C.. (2015). Genome-Wide Investigation of mRNA Lifetime Determinants in Escherichia Coli Cells Cultured at Different Growth Rates. BMC Genomics 16, 275–213. doi: 10.1186/s12864-015-1482-8 PMC4421995 25887031

[B17] Fitzgerald D. M. Bonocora R. P. Wade J. T. (2014). Comprehensive Mapping of the Escherichia Coli Flagellar Regulatory Network. PloS Genet. 10, e1004649. doi: 10.1371/journal.pgen.1004649 25275371 PMC4183435

[B18] Friedlander R. S. Vogel N. Aizenberg J. (2015). Role of Flagella in Adhesion of Escherichia Coli to Abiotic Surfaces. Langmuir 31, 6137–6144. doi: 10.1021/acs.langmuir.5b00815 25945399

[B19] Gassmann M. Grenacher B. Rohde B. Vogel J. (2009). Quantifying Western Blots: Pitfalls of Densitometry. Electrophoresis 30, 1845–1855. doi: 10.1002/elps.200800720 19517440

[B20] Guttenplan S. B. Kearns D. B. (2013). Regulation of Flagellar Motility During Biofilm Formation. FEMS Microbiol. Rev. 37, 849–871. doi: 10.1111/1574-6976.12018 23480406 PMC3718880

[B21] Guzman L. M. Belin D. Carson M. J. Beckwith J. (1995). Tight Regulation, Modulation, and High-Level Expression by Vectors Containing the Arabinose PBAD Promoter. J. Bacteriol. 177, 4121–4130. doi: 10.1128/jb.177.14.4121-4130.1995 7608087 PMC177145

[B22] Heydorn A. Nielsen A. T. Hentzer M. Sternberg C. Givskov M. Ersbøll B. K.. (2000). Quantification of Biofilm Structures by the Novel Computer Program COMSTAT. Microbiol. (Reading Engl.) 146 ( Pt 10), 2395–2407. doi: 10.1099/00221287-146-10-2395 11021916

[B23] Jackson D. W. Suzuki K. Oakford L. Simecka J. W. Hart M. E. Romeo T. (2002). Biofilm Formation and Dispersal Under the Influence of the Global Regulator CsrA of Escherichia Coli. J. Bacteriol. 184, 290–301. doi: 10.1128/JB.184.1.290-301.2002 11741870 PMC134780

[B24] Jefferson K. K. (2004). What Drives Bacteria to Produce a Biofilm? FEMS Microbiol. Lett. 236, 163–173. doi: 10.1111/j.1574-6968.2004.tb09643.x 15251193

[B25] Junker L. M. Peters J. E. Hay A. G. (2006). Global Analysis of Candidate Genes Important for Fitness in a Competitive Biofilm Using DNA-Array-Based Transposon Mapping. Microbiol. (Reading Engl.) 152, 2233–2245. doi: 10.1099/mic.0.28767-0 16849790

[B26] Junker L. M. Toba F. A. Hay A. G. (2007). Transcription in Escherichia Coli PHL628 Biofilms. FEMS Microbiol. Lett. 268, 237–243. doi: 10.1111/j.1574-6968.2006.00585.x 17227468

[B27] Karatan E. Watnick P. (2009). Signals, Regulatory Networks, and Materials That Build and Break Bacterial Biofilms. Microbiol. Mol. Biol. Rev. 73, 310–347. doi: 10.1128/MMBR.00041-08 19487730 PMC2698413

[B28] Katsikogianni M. Missirlis Y. F. (2004). Concise Review of Mechanisms of Bacterial Adhesion to Biomaterials and of Techniques Used in Estimating Bacteria-Material Interactions. Eur. Cell Mater. 8, 37–57. doi: 10.22203/ecm.v008a05 15593018

[B29] Lewandowski Z. Beyenal H. (2013). Fundamentals of Biofilm Research. 2nd (Boca Raton, FL: CRC Press). doi: 10.1201/b16291

[B30] Lewis K. (2001). Riddle of Biofilm Resistance. Antimicrob. Agents Chemother. 45, 999–1007. doi: 10.1128/AAC.45.4.999-1007.2001 11257008 PMC90417

[B31] Li G. W. Burkhardt D. Gross C. Weissman J. S. (2014). Quantifying Absolute Protein Synthesis Rates Reveals Principles Underlying Allocation of Cellular Resources. Cell 157 (3), 624–635. doi: 10.1016/j.cell.2014.02.033 24766808 PMC4006352

[B32] Li C. Louise C. J. Shi W. Adler J. (1993). Adverse Conditions Which Cause Lack of Flagella in Escherichia Coli. J. Bacteriol. 175, 2229–2235. doi: 10.1128/jb.175.8.2229-2235.1993 8385664 PMC204508

[B33] Lindsay D. Holy v. A. (2006). Bacterial Biofilms Within the Clinical Setting: What Healthcare Professionals Should Know. J. Hosp. Infect. 64, 313–325. doi: 10.1016/j.jhin.2006.06.028 17046102

[B34] Liu Z. Niu H. Wu S. Huang R. (2014). CsgD Regulatory Network in a Bacterial Trait-Altering Biofilm Formation. Emerg. Microbes Infect. 3, e1–e1. doi: 10.1038/emi.2014.1 26038492 PMC3913822

[B35] Merritt J. H. Kadouri D. E. O’Toole G. A. (2005). Growing and Analyzing Static Biofilms. Curr. Protoc. Microbiol. 00:1B.1–1B.1.17. doi: 10.1002/9780471729259.mc01b01s00 . PMC4568995 18770545

[B36] Mika F. Hengge R. (2013). Small Regulatory RNAs in the Control of Motility and Biofilm Formation in E. Coli and Salmonella. Int. J. Mol. Sci. 14, 4560–4579. doi: 10.3390/ijms14034560 23443158 PMC3634460

[B37] Morrison R. B. McCapra J. (1961). Flagellar Changes in Escherichia Coli Induced by Temperature of the Environment. Nature 192, 774–776. doi: 10.1038/192774a0

[B38] Mytelka D. S. Chamberlin M. J. (1996). Escherichia Coli fliAZY Operon. J. Bacteriol. 178, 24–34. doi: 10.1128/jb.178.1.24-34.1996 8550423 PMC177617

[B39] Namioka S. Yoshida N. Konno H. Makabe K. (2020). Residue-Specific Binding Mechanisms of Thioflavin T to a Surface of Flat β-Sheets Within a Peptide Self-Assembly Mimic. Biochemistry 59, 2782–2787. doi: 10.1021/acs.biochem.0c00280 32496046

[B40] O’Toole G. Kaplan H. B. Kolter R. (2003). Biofilm Formation as Microbial Development. Annu. Rev. Microbiol. 54, 49–79. doi: 10.1146/annurev.micro.54.1.49 11018124

[B41] Park K. Choi S. Ko M. Park C. (2001). Novel sigmaF-Dependent Genes of Escherichia Coli Found Using a Specified Promoter Consensus. FEMS Microbiol. Lett. 202, 243–250. doi: 10.1111/j.1574-6968.2001.tb10811.x 11520622

[B42] Patel R. (2005). Biofilms and Antimicrobial Resistance. Clin. Orthop. Relat. Res. 437, 41–47. doi: 10.1097/01.blo.0000175714.68624.74 16056024

[B43] Perni S. Preedy E. C. Landini P. Prokopovich P. (2016). Influence of csgD and ompR on Nanomechanics, Adhesion Forces, and Curli Properties of E. Coli. Langmuir 32, 7965–7974. doi: 10.1021/acs.langmuir.6b02342 27434665

[B44] Pesavento C. Becker G. Sommerfeldt N. Possling A. Tschowri N. Mehlis A.. (2008). Inverse Regulatory Coordination of Motility and Curli-Mediated Adhesion in Escherichia Coli. Genes Dev. 22, 2434–2446. doi: 10.1101/gad.475808 18765794 PMC2532929

[B45] Prigent-Combaret C. Brombacher E. Vidal O. Ambert A. Lejeune P. Landini P.. (2001). Complex Regulatory Network Controls Initial Adhesion and Biofilm Formation in Escherichia Coli via Regulation of the csgD Gene. J. Bacteriol. 183, 7213–7223. doi: 10.1128/JB.183.24.7213-7223.2001 11717281 PMC95571

[B46] Prigent-Combaret C. Prensier G. Le Thi T. T. Vidal O. Lejeune P. Dorel C. (2000). Developmental Pathway for Biofilm Formation in Curli-Producing Escherichia Coli Strains: Role of Flagella, Curli and Colanic Acid. Environ. Microbiol. 2, 450–464. doi: 10.1046/j.1462-2920.2000.00128.x 11234933

[B47] Prigent-Combaret C. Vidal O. Dorel C. Lejeune P. (1999). Abiotic Surface Sensing and Biofilm-Dependent Regulation of Gene Expression in Escherichia Coli. J. Bacteriol. 181, 5993–6002. doi: 10.1128/JB.181.19.5993-6002.1999 10498711 PMC103626

[B48] Reichhardt C. Jacobson A. N. Maher M. C. Uang J. McCrate O. A. Eckart M.. (2015). Congo Red Interactions With Curli-Producing E. Coli and Native Curli Amyloid Fibers. PloS One 10, e0140388. doi: 10.1371/journal.pone.0140388 26485271 PMC4618944

[B49] Reisner A. Haagensen J. A. J. Schembri M. A. Zechner E. L. Molin S. (2003). Development and Maturation of Escherichia Coli K-12 Biofilms. Mol. Microbiol. 48, 933–946. doi: 10.1046/j.1365-2958.2003.03490.x 12753187

[B50] Reisner A. Maierl M. Jörger M. Krause R. Berger D. Haid A.. (2014). Type 1 Fimbriae Contribute to Catheter-Associated Urinary Tract Infections Caused by Escherichia Coli. J. Bacteriol. 196 (5), 931 – 939. doi: 10.1128/JB.00985-13 24336940 PMC3957706

[B51] Ren D. Bedzyk L. A. Thomas S. M. Ye R. W. Wood T. K. (2004). Gene Expression in Escherichia Coli Biofilms. Appl. Microbiol. Biotechnol. 64, 515–524. doi: 10.1007/s00253-003-1517-y 14727089

[B52] Schindelin J. Arganda-Carreras I. Frise E. Kaynig V. Longair M. Pietzsch T.. (2012). Fiji: An Open-Source Platform for Biological-Image Analysis. Nat. Methods 9, 676–682. doi: 10.1038/nmeth.2019 22743772 PMC3855844

[B53] Schlafer S. Meyer R. L. (2016). Confocal Microscopy Imaging of the Biofilm Matrix. J. Microbiol. Methods. 138, 50–59. doi: 10.1016/j.mimet.2016.03.002 26979645

[B54] Schneider C. A. Rasband W. S. Eliceiri K. W. (2012). NIH Image to ImageJ: 25 Years of Image Analysis. Nat. Methods 9, 671–675. doi: 10.1038/nmeth.2089 22930834 PMC5554542

[B55] Schwank S. Rajacic Z. Zimmerli W. Blaser J. (1998). Impact of Bacterial Biofilm Formation on *In Vitro* and *In Vivo* Activities of Antibiotics. Antimicrob. Agents Chemother. 42, 895–898. doi: 10.1128/AAC.42.4.895 9559803 PMC105562

[B56] Serra D. O. Richter A. M. Hengge R. (2013). Cellulose as an Architectural Element in Spatially Structured Escherichia Coli Biofilms. J. Bacteriol. 195, 5540–5554. doi: 10.1128/JB.00946-13 24097954 PMC3889604

[B57] Sharma G. Sharma S. Sharma P. Chandola D. Dang S. Gupta S.. (2016). Escherichia Coli Biofilm: Development and Therapeutic Strategies. J. Appl. Microbiol. 121, 309–319. doi: 10.1111/jam.13078 26811181

[B58] Shihan M. H. Novo S. G. Le Marchand S. J. Wang Y. Duncan M. K. (2021). A Simple Method for Quantitating Confocal Fluorescent Images. Biochem. Biophys. Rep. 25:100916. doi: 10.1016/j.bbrep.2021.100916 33553685 PMC7856428

[B59] Shimada T. Tanaka K. Ishihama A. (2017). The Whole Set of the Constitutive Promoters Recognized by Four Minor Sigma Subunits of Escherichia Coli RNA Polymerase. PloS One 12, e0179181. doi: 10.1371/journal.pone.0179181 28666008 PMC5493296

[B60] Singh S. Singh S. K. Chowdhury I. Singh R. (2017). Understanding the Mechanism of Bacterial Biofilms Resistance to Antimicrobial Agents. Open Microbiol. J. 11, 53–62. doi: 10.2174/1874285801711010053 28553416 PMC5427689

[B61] Stewart P. S. (2015). Antimicrobial Tolerance in Biofilms. Microbiol. Spectr. 3 (3), 1–13. doi: 10.1128/microbiolspec.MB-0010-2014 PMC4507308 26185072

[B62] Stewart P. S. Costerton J. W. (2001). Antibiotic Resistance of Bacteria in Biofilms. Lancet 358, 135–138. doi: 10.1016/S0140-6736(01)05321-1 11463434

[B63] Suryaletha K. Narendrakumar L. John J. Radhakrishnan M. P. George S. Thomas S. (2019). Decoding the Proteomic Changes Involved in the Biofilm Formation of Enterococcus Faecalis SK460 to Elucidate Potential Biofilm Determinants. BMC Microbiol. 19, 146. doi: 10.1186/s12866-019-1527-2 31253082 PMC6599329

[B64] Taylor S. C. Posch A. (2014). The Design of a Quantitative Western Blot Experiment. BioMed. Res. Int. 2014, 361590. doi: 10.1155/2014/361590 24738055 PMC3971489

[B65] Vasicek E. M. O’Neal L. Parsek M. R. Fitch J. White P. Gunn J. S. (2021). L-Arabinose Transport and Metabolism in Salmonella Influences Biofilm Formation. Front. Cell Infect. Microbiol. 11:698146. doi: 10.3389/fcimb.2021.698146 34368016 PMC8341724

[B66] Vidal O. Longin R. Prigent-Combaret C. Dorel C. Hooreman M. Lejeune P. (1998). Isolation of an Escherichia Coli K-12 Mutant Strain Able to Form Biofilms on Inert Surfaces: Involvement of a New ompR Allele That Increases Curli Expression. J. Bacteriol. 180, 2442–2449. doi: 10.1128/JB.180.9.2442-2449.1998 9573197 PMC107187

[B67] Vorregaard M. (2008). Comstat2 - a Modern 3D Image Analysis Environment for Biofilms, in Informatics and Mathematical Modelling. Inf. Math. Modelling.

[B68] Wijman J. G. E. de Leeuw P. P. L. A. Moezelaar R. Zwietering M. H. Abee T. (2007). Air-Liquid Interface Biofilms of Bacillus Cereus: Formation, Sporulation, and Dispersion. Appl. Environ. Microbiol. 73, 1481–1488. doi: 10.1128/AEM.01781-06 17209076 PMC1828785

[B69] Wolska K. I. Grudniak A. M. Rudnicka Z. Markowska K. (2016). Genetic Control of Bacterial Biofilms. J. Appl. Genet. 57, 225–238. doi: 10.1007/s13353-015-0309-2 26294280 PMC4830867

[B70] Wong K. K. W. Olsson A. L. J. Asadishad B. van der Bruggen B. Tufenkji N. (2017). Role of Cell Appendages in Initial Attachment and Stability of E. Coli on Silica Monitored by Nondestructive TIRF Microscopy. Langmuir 33, 4066–4075. doi: 10.1021/acs.langmuir.7b00314 28368615

[B71] Yang C.-L. Chen X.-K. Wang R. Lin J.-Q. Liu X.-M. Pang X.. (2019). Essential Role of ς Factor RpoF in Flagellar Biosynthesis and Flagella-Mediated Motility of Acidithiobacillus Caldus. Front. Microbiol. 10:1130. doi: 10.3389/fmicb.2019.01130 31178842 PMC6543871

[B72] Zhang T. Pabst B. Klapper I. Stewart P. S. (2013). General Theory for Integrated Analysis of Growth, Gene, and Protein Expression in Biofilms. Plos One 8, e83626. doi: 10.1371/journal.pone.0083626 24376726 PMC3871705

[B73] Zogaj X. Nimtz M. Rohde M. Bokranz W. Römling U. (2001). The Multicellular Morphotypes of Salmonella Typhimurium and Escherichia Coli Produce Cellulose as the Second Component of the Extracellular Matrix. Mol. Microbiol. 39, 1452–1463. doi: 10.1046/j.1365-2958.2001.02337.x 11260463

