# Supplementary material for: Temperature-Dependent Influence of FliA Overexpression on PHL628 E. coli Biofilm Growth and Composition
Source: Front Cell Infect Microbiol. 2021 Dec 17;11:775270. doi: 10.3389/fcimb.2021.775270 (PMC8718923; doi:10.3389/fcimb.2021.775270)
Supplement: Supplementary file 1 [file DataSheet_1.docx]

**Supplemental Information**

**Temperature-dependent influence of FliA overexpression on PHL628 *E. coli* biofilm growth and composition**

Luke D. Buck, Maddison M. Paladino, Kyogo Nagashima, Emma R. Brezel, Joshua S. Holtzman, Sarel J. Loewus, Lisa M. Ryno^*^

^*^ To whom correspondence should be addressed:

e-mail: [lryno@oberlin.edu](mailto:lryno@oberlin.edu)

Telephone: 440-775-8238

Facsimile: 440-775-6682.

***Table of Contents***

Page

**Table S1:** S3

**Table S2**: S3

**Table S3:**  S4

**Figure S1:** S5

**Figure S2:** S5

**Figure S3:** S6

**Figure S4:** S7

**Figure S5:** S8

**Figure S6:** S9

**Figure S7:** S10

**Figure S8:** S11

**References** ………………………………………………………………………….S12


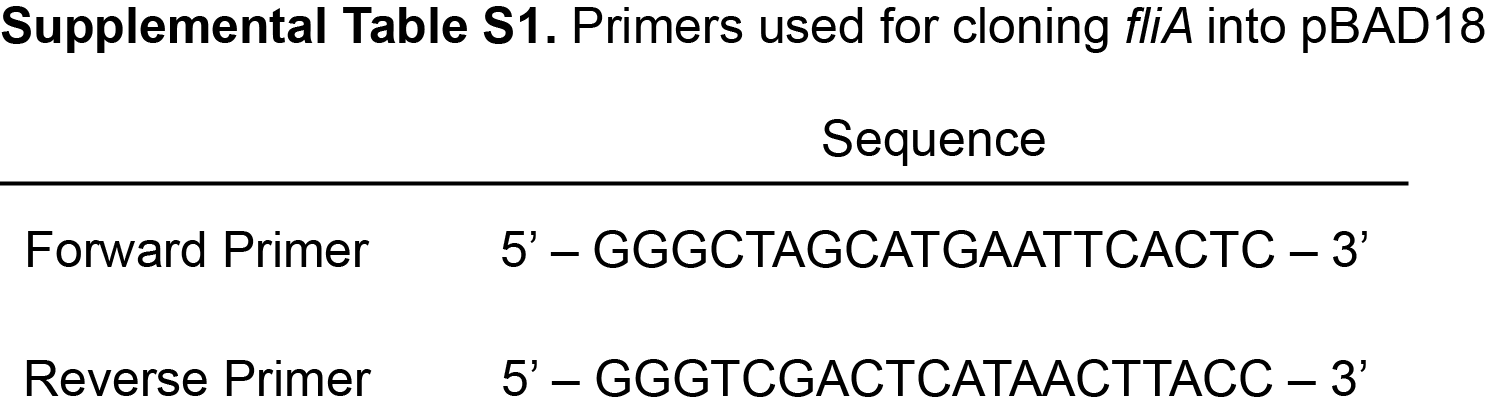


**
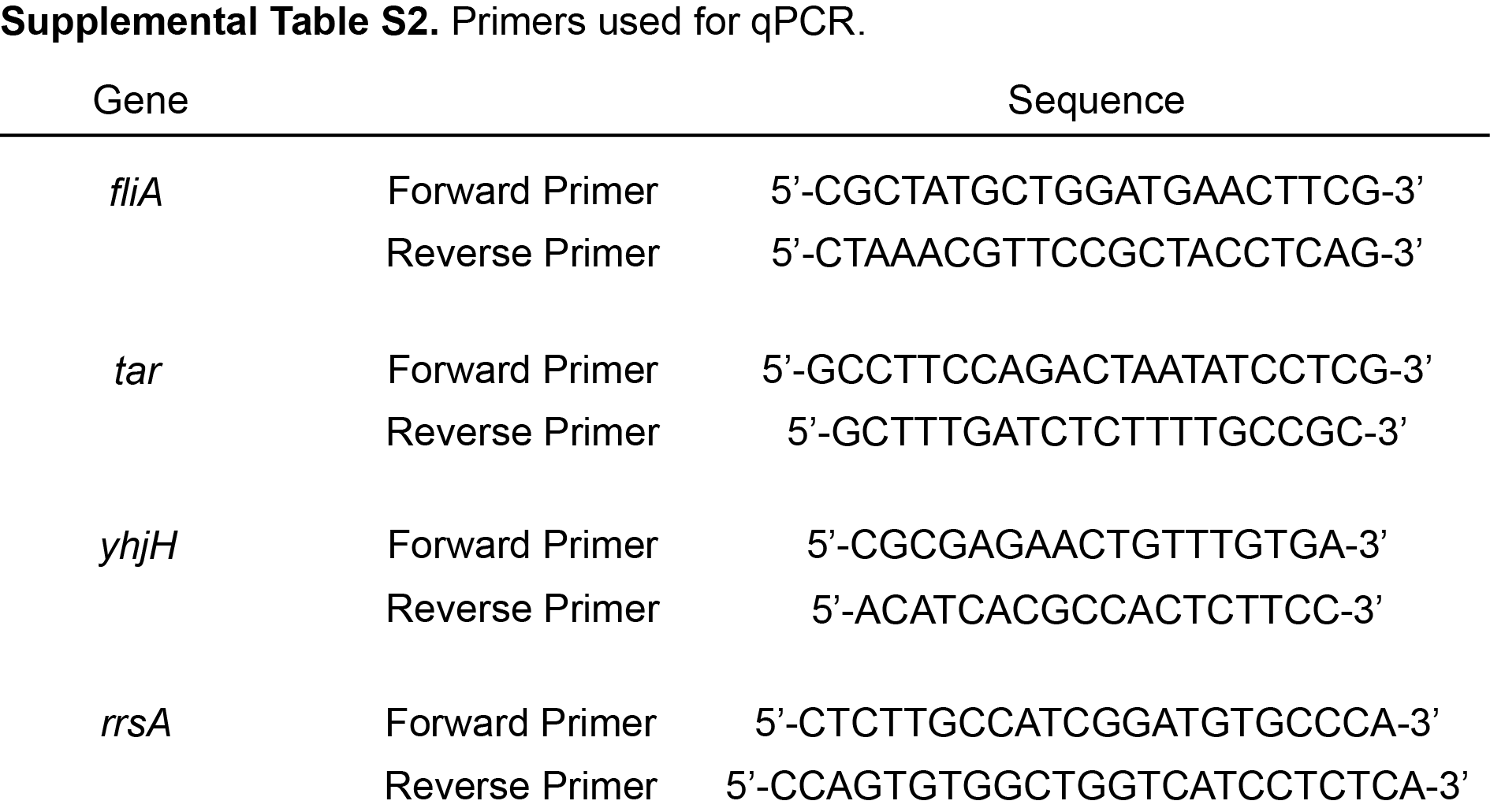
**

**
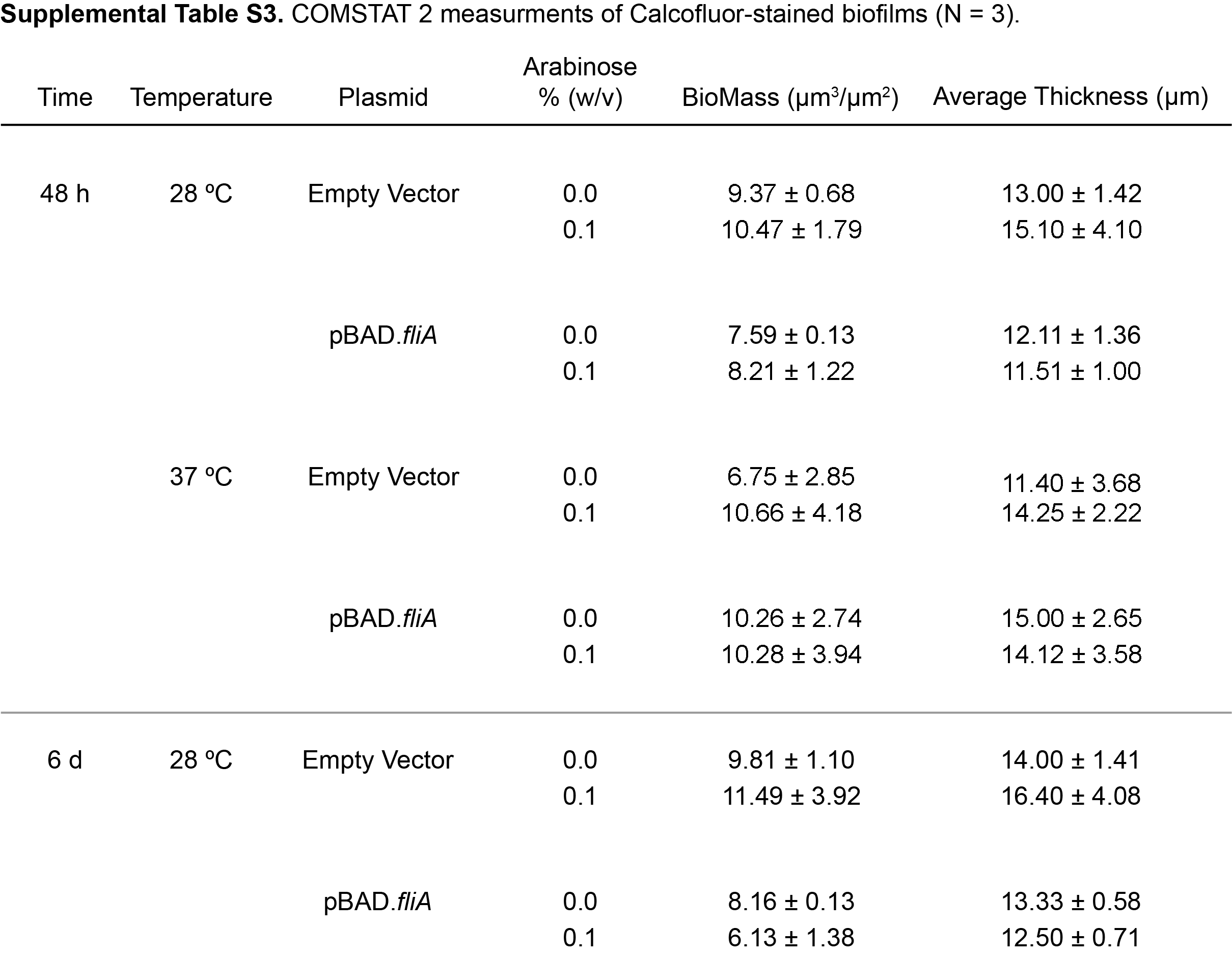
**

**
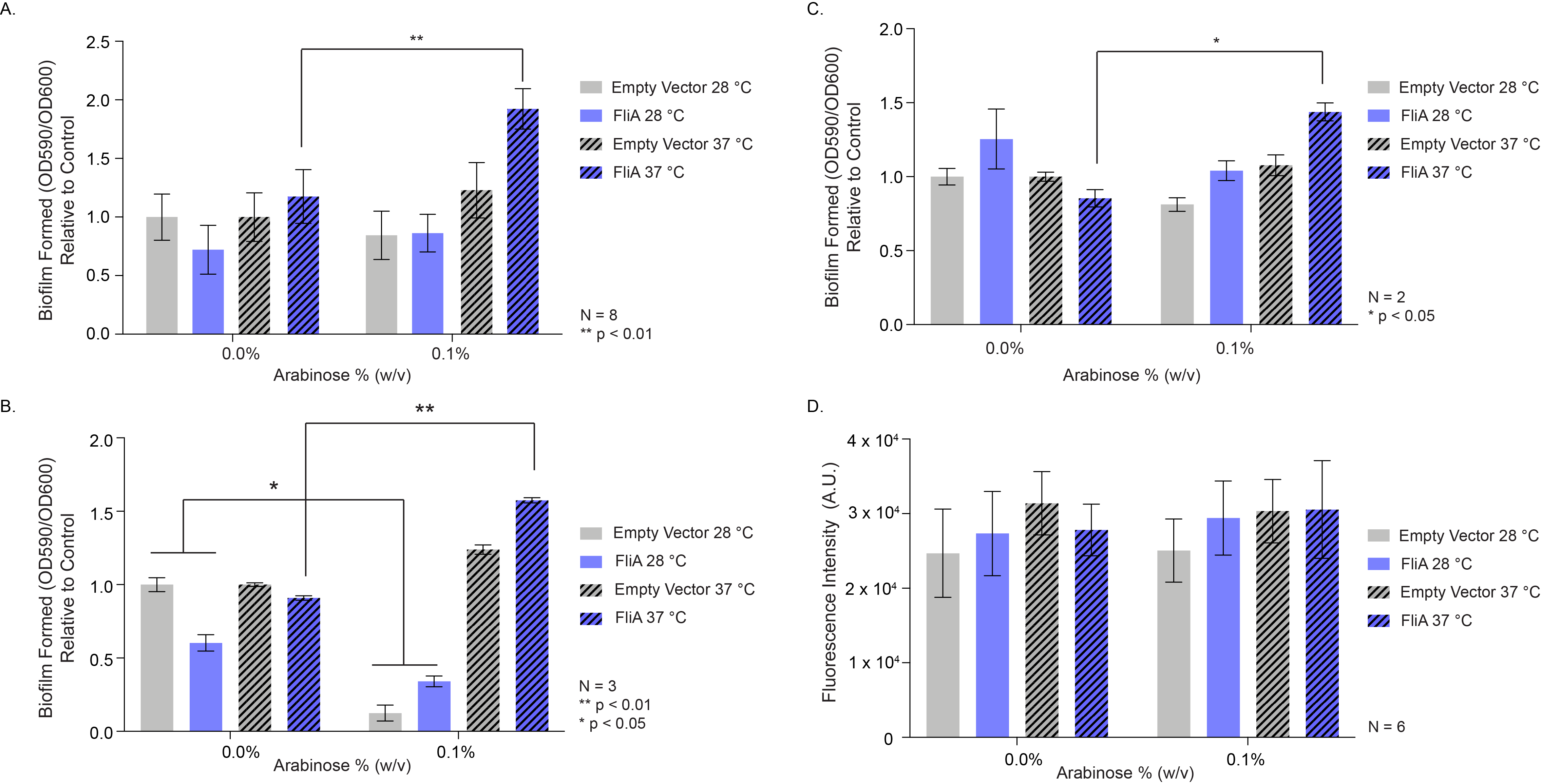
**

**Figure S1.** Amount of biofilm grown in a crystal violet biofilm growth assay after 24 h in **A.** 96-well polystyrene plates, after 48 h on **B.** uncoated or **C.** poly-L-lysine coated silica microscopy slides. **D.** AlamarBlue fluorescence assay of PHL628 cells after 48 hours of growth showing similar viability across all experimental conditions (N =6).

**
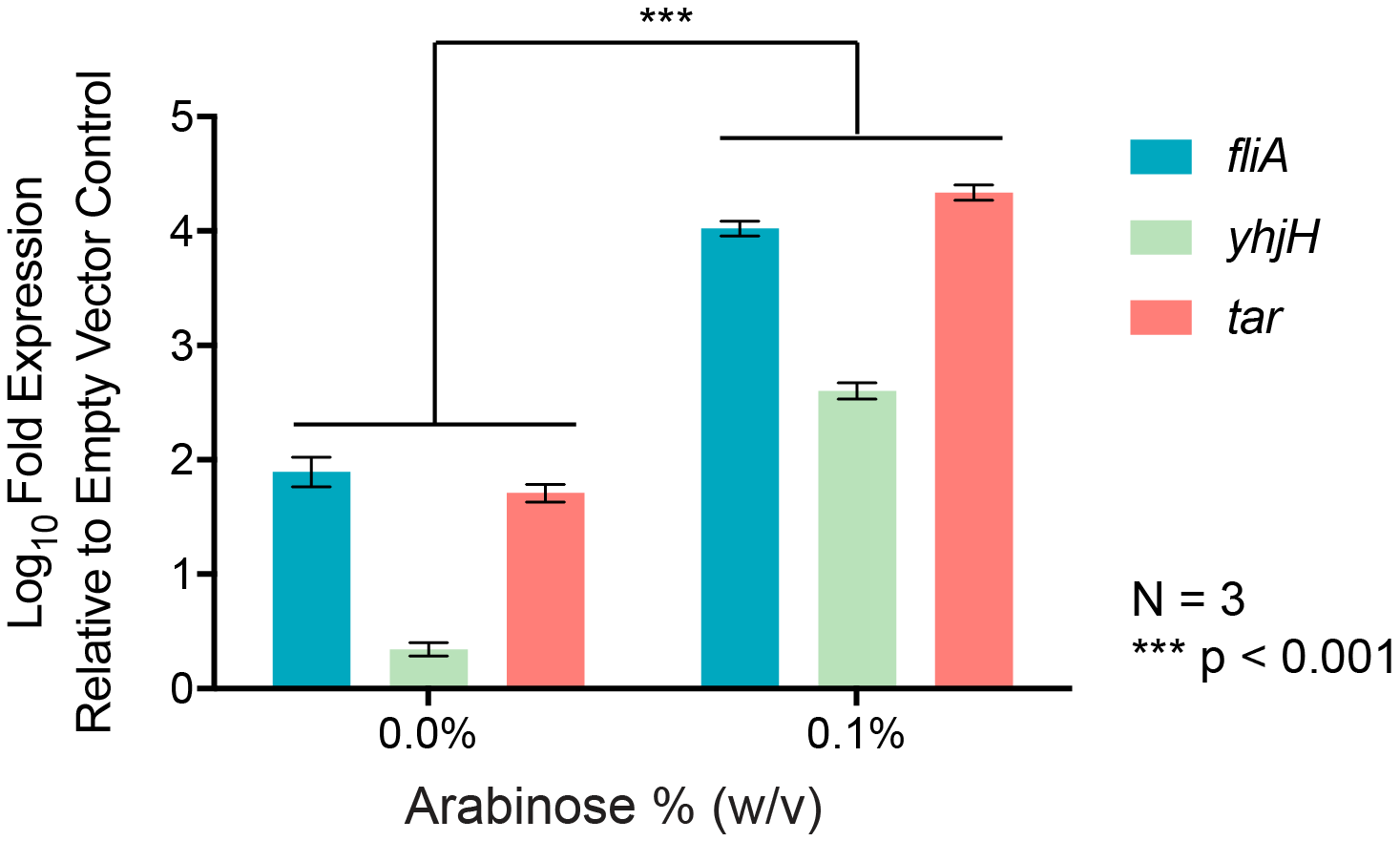
**

**Figure S2.** qPCR results of the pBAD.*fliA* overexpression 10 min after arabinose induction at 37 °C. All data is normalized to that of the empty vector containing *E. coli* cells with 0% arabinose added to them. Significance is reported as standard deviation and analyzed by an unpaired Student’s t-test.

**
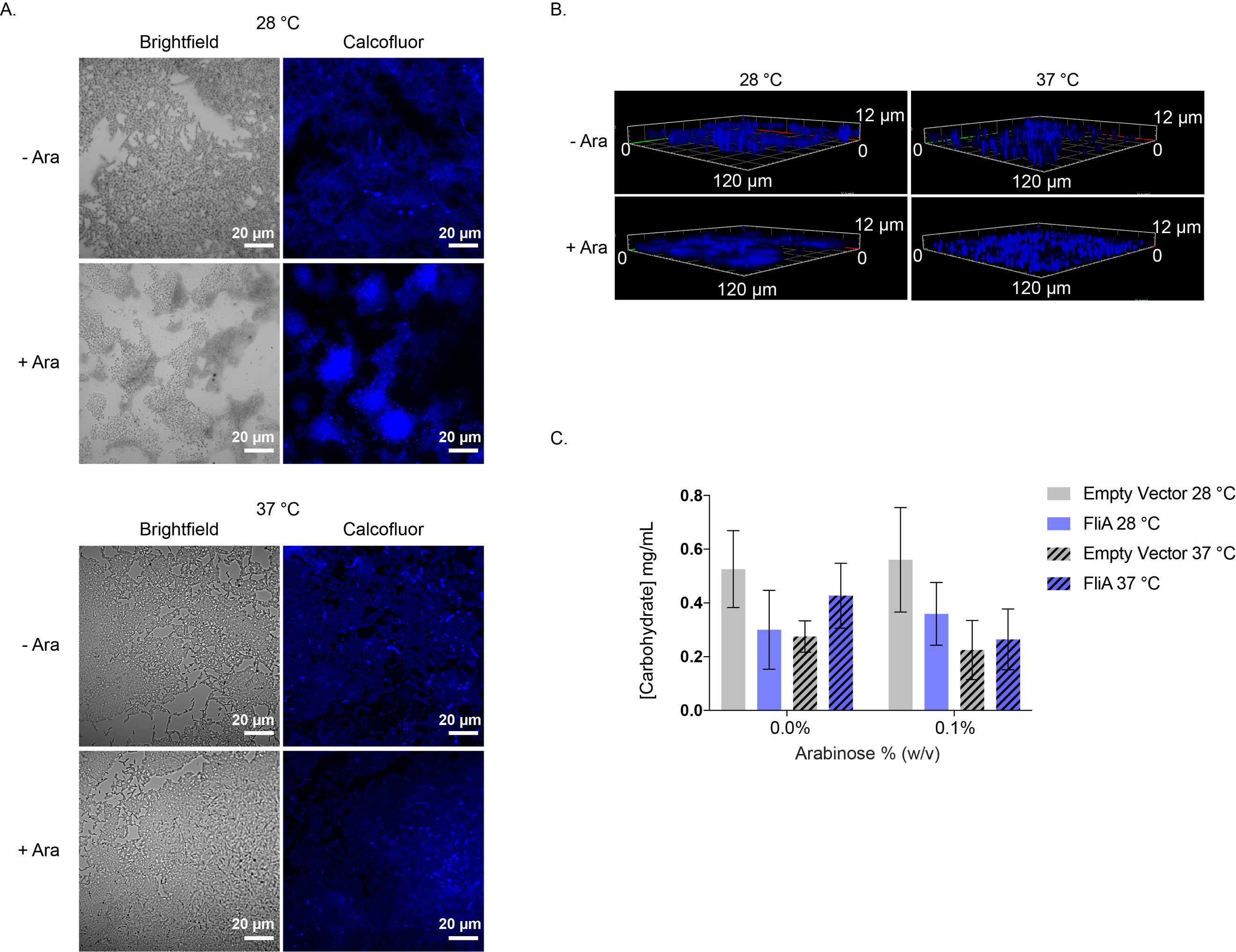
**

**Figure S3.** CLSM Analysis of Calcofluor-stained biofilms. **A.** Representative images of empty vector PHL628 cells under differing experimental conditions (Ara = arabinose) after 48 h growth. Quantitation reported in **Figure 2**. **B.** Representative z-stacks for empty vector PHL628 cells after 6 d growth. **C.** Total carbohydrate concentration of EPS from phenol sulfuric acid assay (N = 3).

**
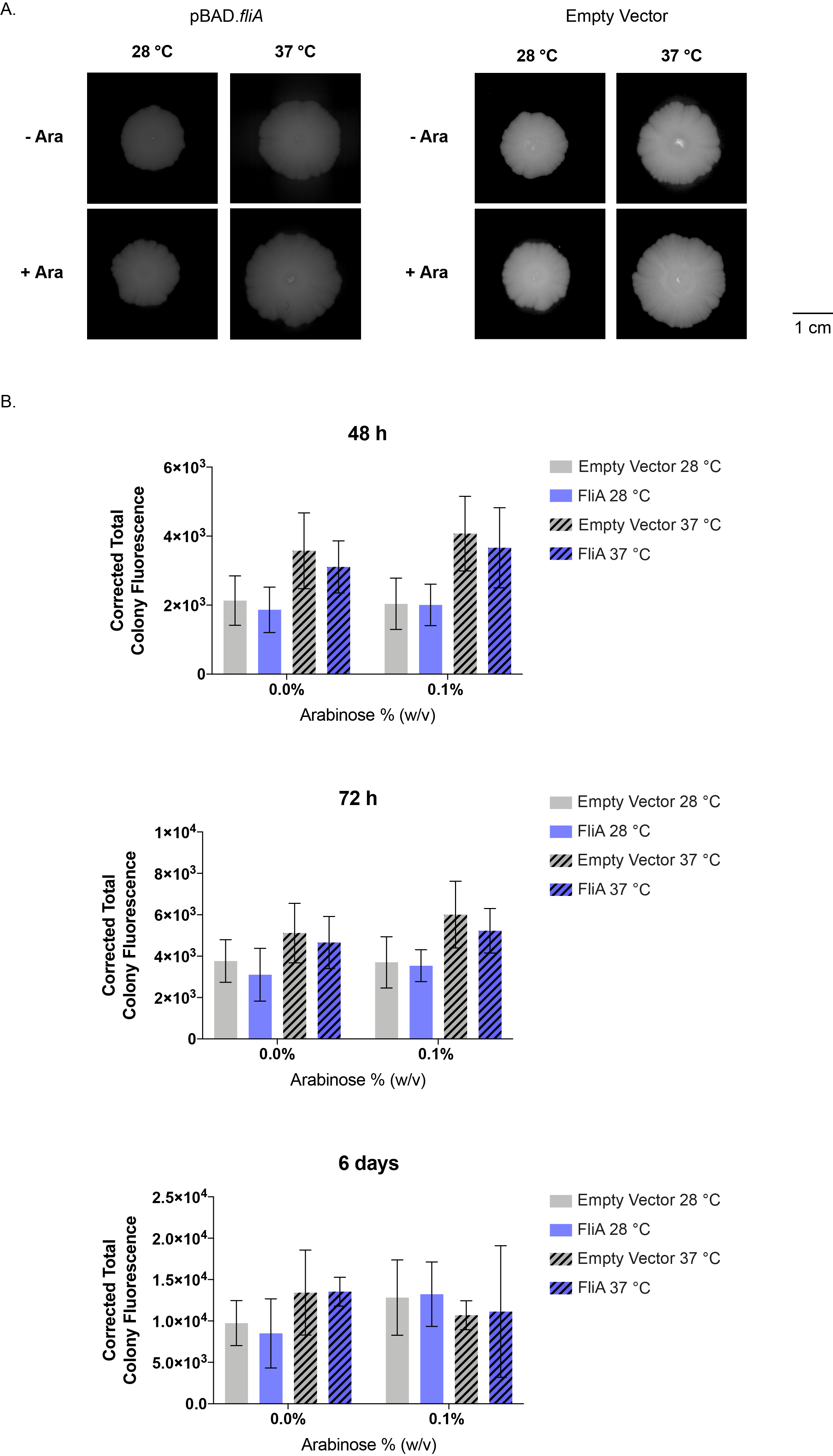
**

**Figure S4.** Calcofluor White Agar Plates**. A.** Representative Calcofluor White agar plates growing pBAD.*fliA* and empty vector PHL628 biofilms for 72 h. Scale bar is equal to 1 cm. Ara = arabinose **B.** Quantification of calcofluor fluorescence using ImageJ (N = 3).

**
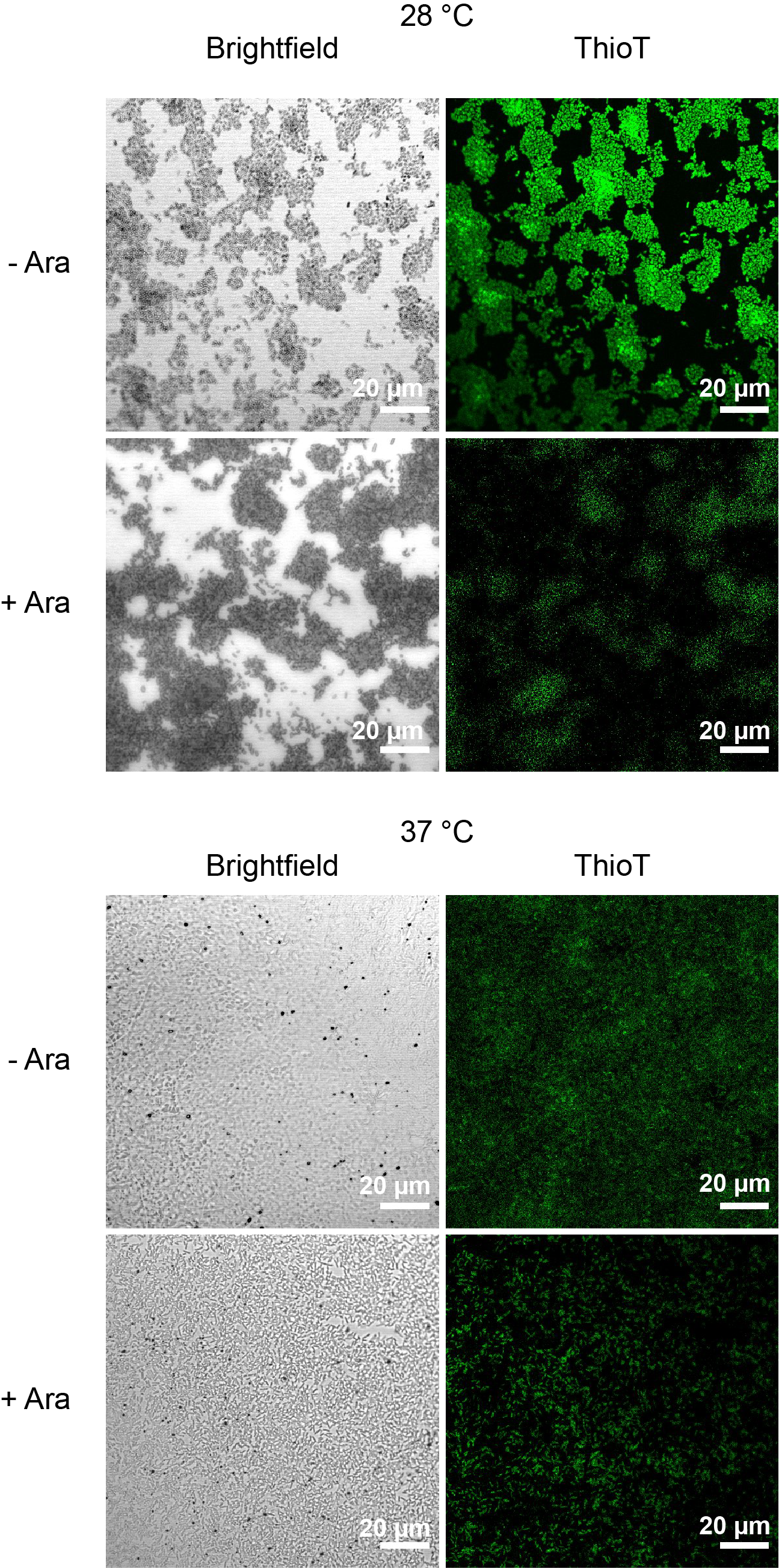
**

**Figure S5.** CLSM Analysis of thioflavin T-stained biofilms. Representative images of empty vector PHL628 cells under differing experimental conditions after 48 h growth. Ara = arabinose.

**
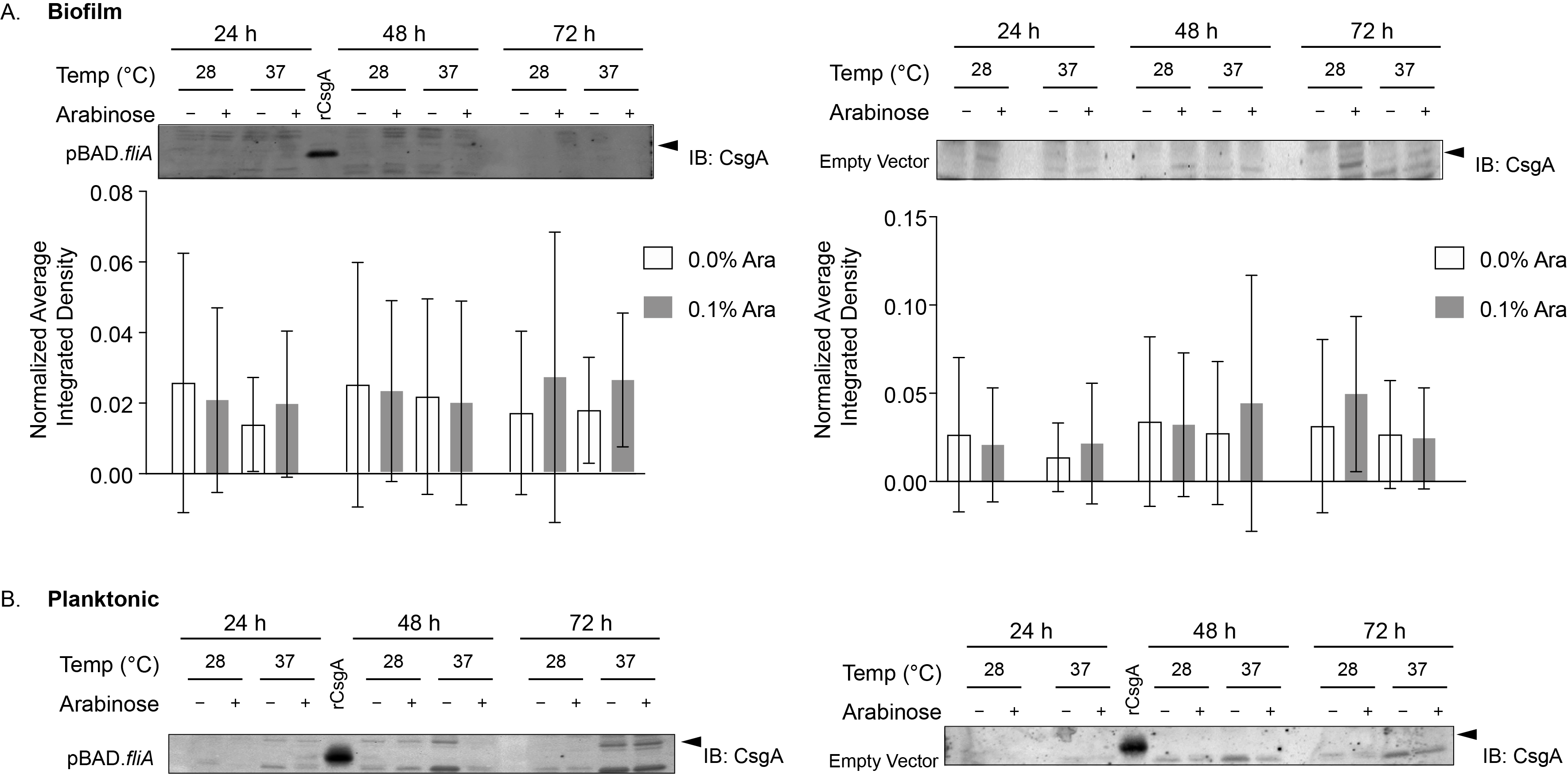
**

**Figure S6.** Immunoblot of biofilm and planktonic cells probed with anti-CsgA antibody.


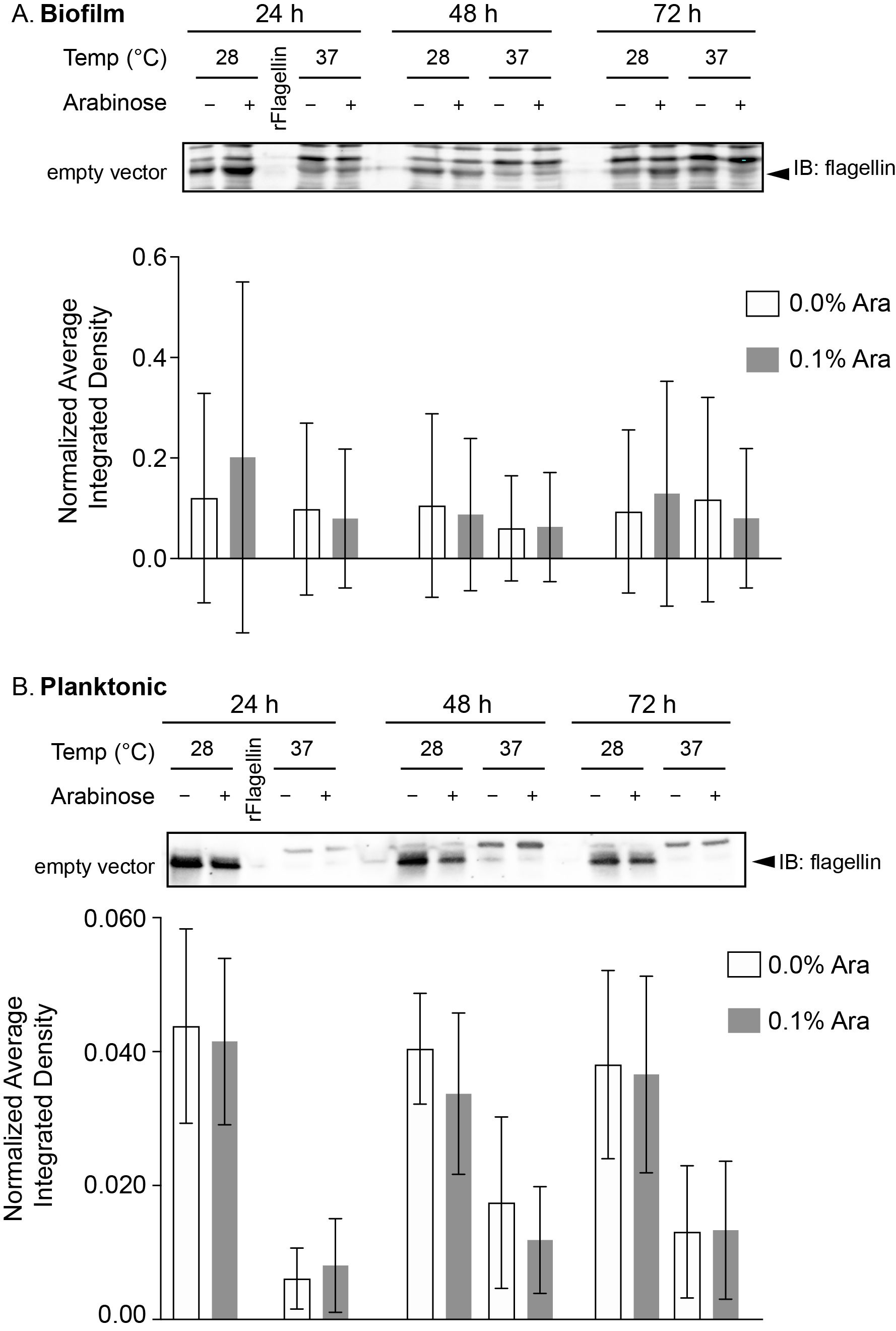


**Figure S7.** Immunoblots and quantitation of empty vector PHL628 **A.** biofilm and **B.** planktonic cells probed with anti-flagellin antibody (N = 3). Ara = arabinose.


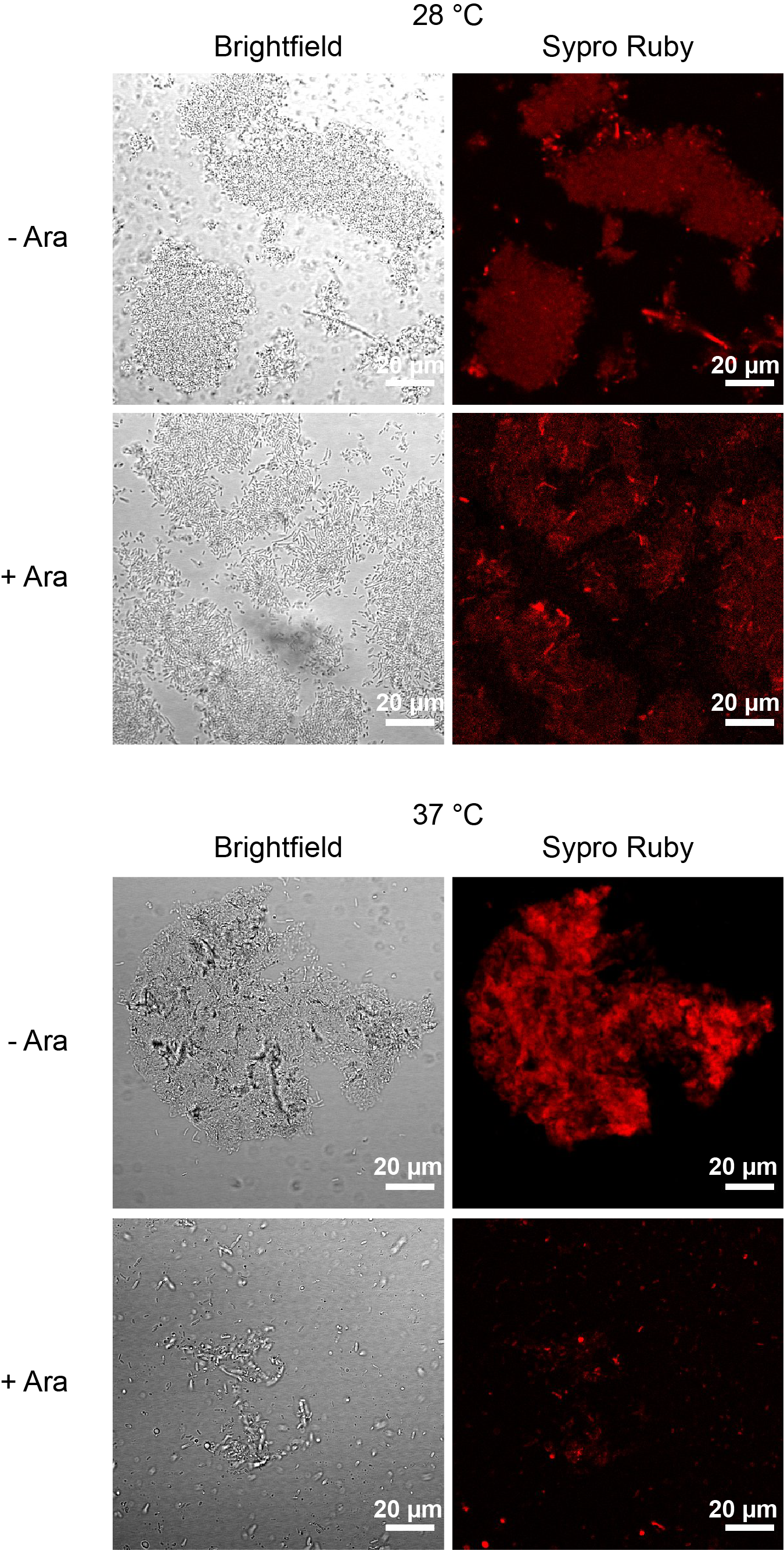


**Figure S8. C**LSM Analysis of Sypro Ruby-stained biofilms. Representative images of empty vector PHL628 cells under differing experimental conditions after 48 h growth. Ara = arabinose

**References**

Li GW, Burkhardt D, Gross C, Weissman JS. (2014). Quantifying absolute protein synthesis rates reveals principles underlying allocation of cellular resources. *Cell*. 157(3):624-635.
